# Degradation of DRAK1 by CUL3/SPOP E3 Ubiquitin ligase promotes tumor growth of paclitaxel-resistant cervical cancer cells

**DOI:** 10.1038/s41419-022-04619-w

**Published:** 2022-02-22

**Authors:** Kyoungwha Pang, Jihee Lee, Junil Kim, Jinah Park, Yuna Park, Eunji Hong, Haein An, Akira Ooshima, Minjung Son, Kyung-Soon Park, Jae-Hyun Cho, Cheol Lee, Yong Sang Song, Kyung-Min Yang, Seong-Jin Kim

**Affiliations:** 1 GILO Institute, GILO Foundation, Seoul, 06668 Republic of Korea; 2 Department of Biomedical Science, College of Life Science, CHA University, Seongnam, Gyeonggi-do 13488 Republic of Korea; 3 School of Systems Biomedical Science, Soongsil University, Seoul, 06978 Republic of Korea; 4 Department of Biomedical Science, College of Life Science, Sungkyunkwan University, Suwon, Gyeonggi-do 16419 Republic of Korea; 5 Department of Obstetrics and Gynecology, Soonchunhyang University Seoul Hospital, Seoul, 04401 Republic of Korea; 6 Department of Pathology, College of Medicine, Seoul National University, Seoul, 03080 Republic of Korea; 7 Cancer Research Institute, College of Medicine, Seoul National University, Seoul, 03080 Republic of Korea; 8 Department of Obstetrics and Gynecology, Seoul National University College of Medicine, Seoul, 03081 Republic of Korea; 9 Medpacto Inc., Seoul, 06668 Republic of Korea

**Keywords:** Cancer therapeutic resistance, Ubiquitylation

## Abstract

Despite favorable responses to initial chemotherapy, drug resistance is a major cause limiting chemotherapeutic efficacy in many advanced cancers. However, mechanisms that drive drug-specific resistance in chemotherapy for patients with advanced cancers are still unclear. Here, we report a unique role of death-associated protein kinase-related apoptosis-inducing kinase 1 (DRAK1) associated with paclitaxel resistance in cervical cancer cells. Interestingly, DRAK1 protein level was markedly decreased in paclitaxel-resistant cervical cancer cells without affecting its mRNA expression, which resulted in an increase in tumor necrosis factor receptor-associated factor 6 (TRAF6) expression, as well as an activation of TRAF6-mediated nuclear factor-kappa B (NF-κB) signaling cascade, thereby promoting tumor progression. DRAK1 depletion markedly increased the chemotherapeutic IC_50_ values of paclitaxel in cervical cancer cells. Ectopic expression of DRAK1 inhibited growth of paclitaxel-resistant cervical cancer cells in vitro and in vivo. Furthermore, DRAK1 was markedly underexpressed in chemoresistant cervical cancer patient tissues compared with chemosensitive samples. We found that DRAK1 protein was destabilized through K48-linked polyubiquitination promoted by the Cullin scaffold protein 3 (CUL3) / speckle-type POZ (poxvirus and zinc finger protein) protein (SPOP) E3 ubiquitin ligase in paclitaxel-resistant cells. Collectively, these findings suggest that DRAK1 may serve as a potential predictive biomarker for overcoming paclitaxel resistance in cervical cancer.

## Introduction

Taxane-based chemotherapy is chosen as a single agent or combination with cisplatin to treat patients with recurrent or advanced cervical cancer; however, cancer resistance is an emerging problem that remains to be solved [1, 2]. Although much progress has been made in understanding the mechanisms of cellular resistance, including the expression of multidrug resistance proteins, reduced drug accumulation, enhanced DNA repair, aberrant apoptosis pathways, and altered cell proliferation, there has been no significant biomarkers discovered to predict the recurrence of cervical cancer [3–5]. Thus, it is critical to investigate promising therapeutic targets to overcome cervical cancer chemoresistance to address this challenge.

The nuclear factor-kappa B (NF-κB) signaling pathway is activated by chemotherapy drugs, such as platinum-based anticancer drugs, anthracyclines, and taxanes, and its induction impinges on cellular resistance to anticancer agents [6–8]. Tumor necrosis factor receptor-associated factor 6 (TRAF6), a member of the TRAF family, is known as a key activator of NF-κB signaling by recruiting NF-κB-inducing kinase through ubiquitination [9–11]. Although the function of TRAF6 in inflammation and cancer progression has been widely studied, its role in paclitaxel sensitivity remains to be examined.

DRAK1, DAPK-related apoptosis-inducing protein kinase 1, is a member of the death-associated protein kinase (DAPK) family, also known as STK17A. Our previous study revealed that depletion of DRAK1 enhances the protein expression levels of TRAF6, eventually activating NF-κB signaling and promoting tumorigenesis and lung metastasis in cervical cancer cells [12]. On the other hand, we previously reported that DRAK1 overexpression enhanced tumorigenic potential through the inhibition of TGF-β1 tumor suppressor activity in head and neck cancer cells [13]. In recent studies, the expression of DRAK1 was found to be downregulated in the acquired resistance phenotype of colon cancer, melanoma, pancreatic cancer, and ovarian cancer cells [14–17]. Furthermore, upregulation of DRAK1 expression increased the sensitivity of OVCAR3 cells to carboplatin and paclitaxel; however, the molecular mechanism of this phenomenon is undetermined [16].

Cullin-RING ubiquitin ligases (CRLs) are multimeric complexes with a catalytic center that transfer ubiquitins to their substrates. Recently, it has been shown that the CRL3 subfamily of E3 ubiquitin ligases is involved in the regulation of various human diseases, such as neurodegeneration and cancer. Similar to other CRL family complexes, CRL3 consists of Cullin scaffold protein 3 (CUL3), the RING protein Rbx1, and a variable BTB domain adaptor protein, which recognizes and recruits substrates for ubiquitination [18]. Speckle-type POZ (poxvirus and zinc finger protein) protein (SPOP) is a CRL3 family adaptor protein that plays a critical role in tumorigenesis and progression via ubiquitination-mediated degradation of multiple substrates in kidney cancer [19].

In this study, we show that downregulation of DRAK1 expression is associated with paclitaxel resistance in cervical cancer cells and that DRAK1 protein is degraded by CUL3/SPOP E3 ubiquitin ligase through K48-linked polyubiquitination-mediated proteasomal degradation in paclitaxel-resistant cells, resulting in an increase in TRAF6 levels and TRAF6-mediated NF-κB activation, thereby promoting tumor progression.

## Results

### Loss of DRAK1 protein is associated with paclitaxel resistance in cervical cancer cells

**Fig. 1:**
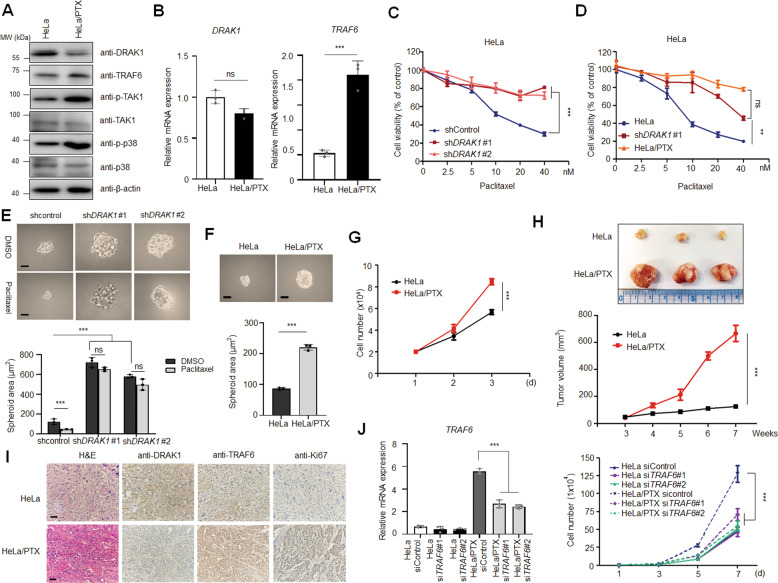
Loss of DRAK1 protein is associated with paclitaxel resistance in cervical cancer cells. **A** Immunoblot analysis of parental HeLa and paclitaxel-resistant HeLa (HeLa/PTX) cells with indicated antibodies and β-actin was used for internal control. **B** qRT-PCR of *DRAK1* and *TRAF6* expressions in HeLa and HeLa/PTX cells and *18* *s* was used for internal control. The data represent the mean ± S.D. of three independent experiments. ns nonsignificant *p < 0.05; **p < 0.01; ***p < 0.001. **C** Cell viability assay of *DRAK1*-knockdowned HeLa cells upon paclitaxel treatment ranging from 2.5 to 40 nM. The data represent the mean ± S.D. of three independent experiments. *p < 0.05; **p < 0.01; ***p < 0.001. **D** Cell viability assay of *DRAK1*-knockdowned HeLa cells and HeLa/PTX with parental HeLa cells upon paclitaxel treatment ranging from 2.5 to 40 nM. The data represent the mean ± S.D. of three independent experiments. ns nonsignificant *p < 0.05; **p < 0.01; ***p < 0.001. **E** Spheroid formation assay of *DRAK1*-knockdowned HeLa cells upon paclitaxel treatment (10 nM). Original magnification 100x. Scale bar, 20 μm. The data represent the mean ± S.D. of three independent experiments. ns non-significant *p < 0.05; **p < 0.01; ***p < 0.001. **F** Spheroid formation assay of HeLa and HeLa/PTX cells. Original magnification 100x. Scale bar, 20 μm. The data represent the mean ± S.D. of three independent experiments. *p < 0.05; **p < 0.01; ***p < 0.001. **G** Cell doublings of HeLa and HeLa/PTX cells. The data represent the mean ± S.D. of three independent experiments. *p < 0.05; **p < 0.01; ***p < 0.001. **H** In vivo tumor-formation assay of HeLa and HeLa/PTX cells subcutaneously injected into the flanks of NRGA mice (*n* = 8 per group). Primary tumor volumes were measured weakly starting 3 weeks after injection and mice were sacrificed at 8weeks. Representative primary tumor images (top) and tumor volumes (bottom) are shown. Error bars represent the mean ± S.D. of independent experiments. *p < 0.05; **p < 0.01; ***p < 0.001. **I** Representative IHC image showing H&E, DRAK1, TRAF6, and Ki67 expression in primary tumor tissues from H. Original magnification 100x. Scale bar, 100 μm. **J** qRT-PCR of *TRAF6* expressions showing *TRAF6*-knockdowned efficiency in HeLa and HeLa/PTX cells (left) and cell doublings of *TRAF6*-knockdowned HeLa and HeLa/PTX cells (right). The data represent the mean ± S.D. of three independent experiments. *p < 0.05; **p < 0.01; ***p < 0.001.

Although it is already known that NF-κB activation by anticancer agents leads to the chemoresistance of cancer cells, the function of TRAF6 associated with drug resistance in cervical cancer has not been elucidated. Considering that loss of DRAK1 expression activates the TRAF6-mediated inflammatory response in metastatic cervical cancer cells reported in our previous study, we examined whether aberrant expression of DRAK1 is related to paclitaxel resistance, which results in tumor progression of cervical cancer cells. First, we generated HeLa cells resistant to paclitaxel by exposing them to stepwise escalating paclitaxel doses for several months and examined the expression levels of DRAK1 and TRAF6 in paclitaxel-resistant HeLa cells (HeLa/PTX). Through immunoblot analysis, we found that DRAK1 expression was suppressed in HeLa/PTX cells compared to parental HeLa cells, whereas TRAF6 expression and that of its downstream signaling proteins, such as p-TAK1 and p-p38, were upregulated (Fig. 1A; Supplementary Fig. S1). Interestingly, the mRNA expression of TRAF6 was upregulated in addition to its protein level, whereas the DRAK1 mRNA level remained unchanged (Fig. 1B), suggesting that DRAK1 protein expression was specifically downregulated in HeLa/PTX cells. In addition, the enhanced activity of the NF-κB promoter after upregulation of TRAF6 expression was investigated in HeLa/PTX cells (Supplementary Fig. S2). To further confirm that DRAK1 protein expression is associated with paclitaxel resistance in cervical cancer cells, we examined the viability of DRAK1-knockdown HeLa and CaSki cell lines after PTX treatment. DRAK1 knockdown efficiency of HeLa and CaSki cell lines was determined by immunoblot analysis (Supplementary Fig. S3A). Notably, DRAK1 depletion markedly increased the chemotherapeutic IC_50_ values of paclitaxel in HeLa (Fig. 1C) and CaSki cells (Supplementary Fig. S3B). The level of increased PTX resistance of DRAK1-depleted HeLa cells was similar to that of HeLa/PTX cells, suggesting that DRAK1 expression is associated with paclitaxel chemoresistance in cervical cancer cells (Fig. 1D). To date, three-dimensional cell culture models, such as spheroids, are preferred for evaluating drug efficacy as they mimic the therapeutic resistance of human solid tumors [20]. We observed an increase in the 3D spheroid formation of DRAK1-depleted HeLa (Fig. 1E) and CaSki cells (Supplementary Fig. S3C) with respect to each control cells. Spheroid formation of DRAK1-depleted HeLa and CaSki cells were not affected by paclitaxel treatment, in contrast to that observed in each parental spheroid.

Next, we observed the phenotypic change of HeLa/PTX cells by performing 3D cell culture and cell proliferation assays with parental HeLa and HeLa/PTX cells. As expected, spheroid formation and the cell proliferation rate were markedly enhanced in HeLa/PTX cells (Fig. 1F, G). To further verify the effect of paclitaxel resistance on tumorigenesis in vivo, we injected parental HeLa and HeLa/PTX cells subcutaneously into the flanks of immunodeficient mice. In accordance with in vitro observations, paclitaxel resistance dramatically increased tumor formation in vivo (Fig. 1H). Also, through immunohistochemistry (IHC) staining of xenograft tissues, we observed that the expression level of DRAK1 was downregulated, whereas those of TRAF6 and Ki67, a marker of cell proliferation, were upregulated in paclitaxel-resistant tissues (Fig. 1I), suggesting that the suppression of DRAK1 protein level and induction of TRAF6 expression were related to the tumor progression of HeLa/PTX cells. As TRAF6 levels were induced in HeLa/PTX cells, we next investigated whether knockdown of TRAF6 resulted in attenuation of the cell proliferation rate of HeLa/PTX cells. *TRAF6* depletion by siRNA transfection in parental HeLa cells had no effect on cell number; however, *TRAF6-*knockdown in HeLa/PTX cells decreased cell proliferation, similar to that of control parental cells (Fig. 1J), suggesting that an increase in TRAF6 levels in HeLa/PTX cells resulted in the induction of cell proliferation. Furthermore, we examined the expressions of genes *ABCB1, IL-1β*, and *IL-8* in HeLa/PTX cells, the NF-κB target genes, which were enhanced in DRAK1-knockdown HeLa cells in our previous study [12]. Indeed, quantitative real-time PCR (qRT-PCR) showed that expression of these target genes was enhanced in HeLa/PTX cells compared to parental HeLa cells and were significantly reduced by knockdown of *TRAF6* (Supplementary Fig. S4). Collectively, these results suggest that a decrease in DRAK1 protein level is associated with paclitaxel resistance in cervical cancer cells and results in an increase in TRAF6 expression and TRAF6-mediated NF-κB activation, promoting tumorigenesis of paclitaxel-resistant cells.

### DRAK1 is significantly underexpressed in chemoresistant cervical cancers

**Fig. 2:**
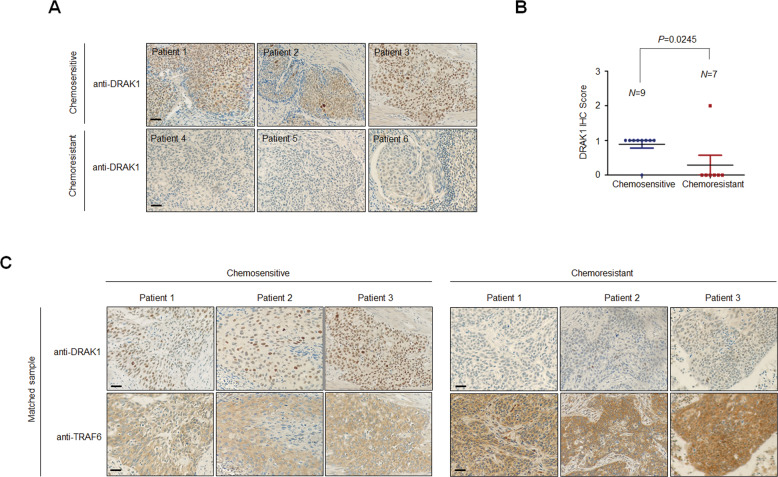
DRAK1 is significantly underexpressed in chemoresistant cervical cancers. **A** Representative IHC image showing the expression of DRAK1 in chemosensitive or chemoresistant cervical cancer patient TMAs. Original magnification 200x. Scale bar, 50 μm. **B** Scatter plots showing the IHC score of DRAK1 from A. *P* values were calculated by paired two-tailed Student’s *t* tests. **C** Representative IHC image showing the expression of DRAK1 and TRAF6 in matched samples from TMAs. Original magnification 200x. Scale bar, 50 μm.

To further evaluate the clinical significance of DRAK1 expression associated with chemoresistance, we performed IHC using human cervical cancer tissue microarrays (TMAs) obtained from Seoul National University College of Medicine in South Korea. We analyzed TMAs of advanced cervical cancer patients who received paclitaxel and cisplatin combination chemotherapy. We classified the patient tissues into two groups as follows: a chemosensitive group, with no disease progression or disease progression more than six months after completion of chemotherapy, and a chemoresistant group, defined as disease progression within six months of completing chemotherapy. Interestingly, DRAK1 expression was remarkably suppressed in tissues of the chemoresistant group (*N* = 9) compared with those of the chemosensitive group (*N* = 7). Moreover, DRAK1 immunostaining score was significantly lower in the chemoresistant group (*p* = 0.0245) (Fig. 2A, B). Next, we observed the correlation between DRAK1 and TRAF6 expression in matched tumor tissue samples. IHC staining of chemoresistant tissues revealed a decreased DRAK1 expression and relatively increased TRAF6 expression in matched patient samples compared to those of chemosensitive tissues (Fig. 2C).

### DRAK1 overexpression decreases the proliferation and tumorigenesis of paclitaxel-resistant cells

**Fig. 3:**
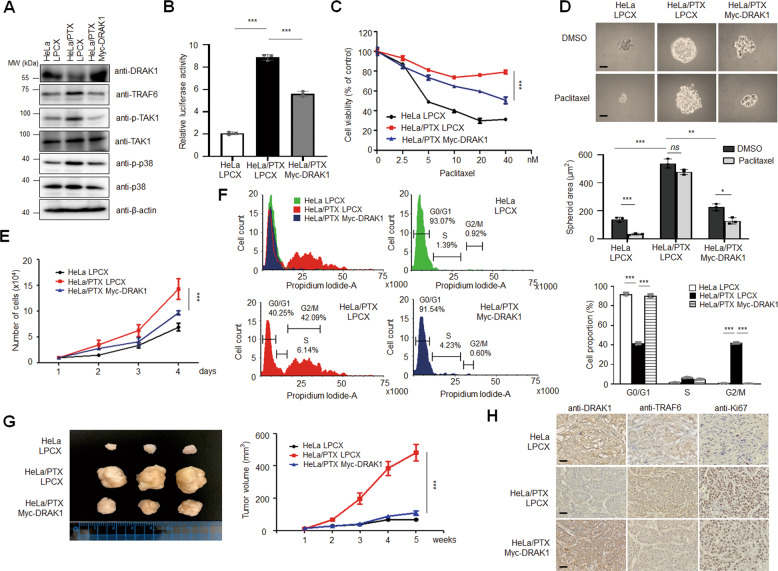
DRAK1 overexpression decreases the cell proliferation and tumorigenesis of paclitaxel-resistant cells. **A** Immunoblot analysis of DRAK1-overexpressing HeLa/PTX cells, HeLa LPCX and HeLa/PTX LPCX cells with indicated antibodies and β-actin was used for internal control. **B** Luciferase activities of NF-κB promoter in DRAK1-overexpressing HeLa/PTX cells, HeLa LPCX, and HeLa/PTX LPCX cells. The data represent the mean ± S.D. of three independent experiments. *p < 0.05; **p < 0.01; ***p < 0.001. **C** Cell viability of DRAK1-overexpressing HeLa/PTX cells upon paclitaxel treatment ranging from 2.5 to 40 nM. The data represent the mean ± S.D. of three independent experiments. *p < 0.05; **p < 0.01; ***p < 0.001**. D** Spheroid formation of DRAK1-overexpressing HeLa/PTX cells, HeLa/PTX LPCX, and HeLa/PTX LPCX cells upon paclitaxel treatment (10 nM). Original magnification 100x. Scale bar, 20 μm. The data represent the mean ± S.D. of three independent experiments. ns nonsignificant *p < 0.05; **p < 0.01; ***p < 0.001. **E** Cell doublings of DRAK1-overexpressing HeLa/PTX cells compared with HeLa/PTX LPCX and HeLa LPCX cell lines. The data represent the mean ± S.D. of three independent experiments. *p < 0.05; **p < 0.01; ***p < 0.001. **F** Cell cycle analysis of DRAK1-overexpressing HeLa/PTX cells compared to HeLa/PTX LPCX and HeLa LPCX using propidium iodide staining. Representative flow histograms visualized the cell cycle transition for each cell lines and percentages of cells in G0/G1, S and G2/M for each cell lines were shown in bar graph. Experiments were performed in triplicate. Data represent means ± S.D. of three independent experiments. *p < 0.05; **p < 0.01; ***p < 0.001. **G** Tumor formation and growth of HeLa LPCX, HeLa/PTX LPCX and DRAK1-overexpressing HeLa/PTX cells. Primary tumor volumes were measured weakly, and mice were sacrificed at 8 weeks (*n* = 5 per group). Representative primary tumor images (left) and tumor volumes (right) are shown. Data represent means ± S.D. of independent experiments. *p < 0.05; **p < 0.01; ***p < 0.001. **H** Representative IHC image showing DRAK1, TRAF6, and Ki67 expression in primary tumor tissues from **G**. Original magnification 100x. Scale bar, 100 μm.

Our findings led us to investigate whether the expression of DRAK1 directly influences the tumorigenic potential of paclitaxel-resistant cells. To this end, we generated a DRAK1-overexpressing HeLa/PTX cell line to determine the effects of ectopically overexpressed DRAK1 on TRAF6 levels and downstream signaling pathway. As expected, overexpression of DRAK1 downregulated TRAF6 protein levels and downregulated the activation of TAK1 and p38 MAPK, suggesting that DRAK1 specifically inhibits the TRAF6-dependent TAK1/p38 MAPK signaling cascade and NF-κB activity in HeLa/PTX cells (Figs. 3A; Supplementary Fig. S3B and S5). Furthermore, DRAK1 overexpression decreased the paclitaxel IC_50_ values of HeLa/PTX LPCX (Fig. 3C). Enhanced spheroid formation of HeLa/PTX LPCX cells was significantly reduced by DRAK1 overexpression, and the anticancer effect of paclitaxel was observed in DRAK1-overexpressing HeLa/PTX cells as in HeLa/PTX LPCX (Fig. 3D). As the cell proliferation rate of HeLa/PTX cells was reduced by TRAF6 knockdown in previous observations, we performed a cell proliferation assay and 3D cell culture assay using HeLa, HeLa/PTX, and DRAK1-overexpressing HeLa/PTX cells. Indeed, DRAK1 overexpression attenuated cell growth of HeLa/PTX LPCX cells (Fig. 3E). Our results raised the possibility that DRAK1 overexpression induces cell cycle arrest in paclitaxel-resistant cells. To test this hypothesis, we performed cell cycle analysis by flow cytometry with propidium iodide staining. The ratio of HeLa/PTX cells in G2/M phase was significantly increased compared to that in parental HeLa cells, as expected (HeLa LPCX: 0.92%, HeLa/PTX LPCX: 42.09%), and the ratio of G0/G1 phase was decreased; however, DRAK1 overexpression dramatically increased the ratio of G0/G1 phase cells compared to the level of HeLa/PTX cells in G0/G1 (HeLa/PTX Myc-DRAK1: 91.54%, HeLa/PTX: 40.25%) and decreased the ratio of G2/M phase cells (HeLa/PTX Myc-DRAK1: 0.60%) (Fig. 3F). On the basis of these in vitro results, to further examine the effect of DRAK1 on the tumorigenic capacity of HeLa/PTX cells in vivo, we subcutaneously injected DRAK1-overexpressing HeLa/PTX cells into the flanks of immunodeficient mice. DRAK1-overexpressing HeLa/PTX cells exhibited an attenuated tumor volume compared to HeLa/PTX cells (Fig. 3G). Intriguingly, the markedly increased expression of TRAF6 and Ki67 in HeLa/PTX tumor tissues was inhibited by DRAK1 overexpression (Fig. 3H), indicating that DRAK1 overexpression inhibits tumor progression by negatively regulating TRAF6 expression. Taken together, these results suggest that DRAK1 acts as a tumor suppressor in TRAF6-mediated tumorigenesis of paclitaxel-resistant cells.

### Overexpression of DRAK1 downregulates the expression of cell cycle-promoting genes in paclitaxel-resistant cells

**Fig. 4:**
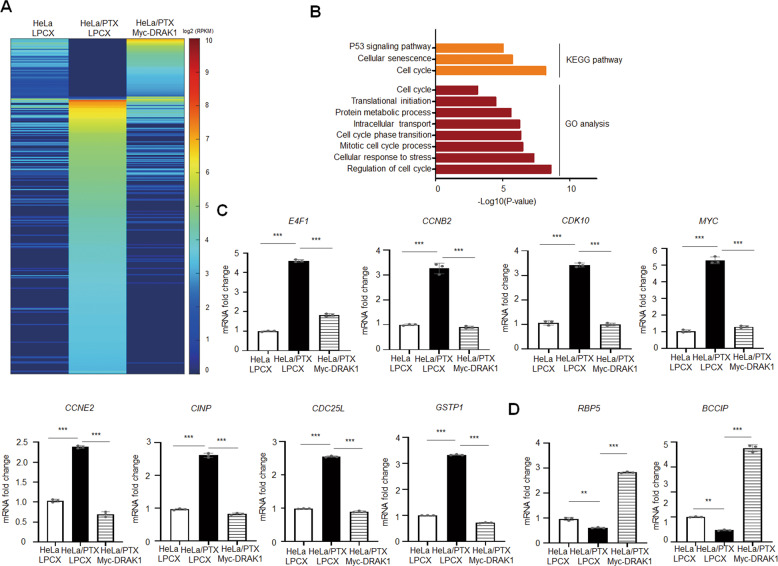
Overexpression of DRAK1 downregulates the expression of cell cycle-promoting genes in paclitaxel-resistant cells. **A** Heatmap showing differential gene expression in DRAK1-overexpressing HeLa/PTX compared to HeLa LPCX or HeLa/PTX LPCX cells. Red represents significantly upregulated genes and blue represents significantly downregulated genes. Threshold values are as follows: corrected value *P* < 0.05 and absolute log2-fold change >1.0. **B** KEGG pathways and GO terms enriched in differentially expressed genes by DRAK1 expression from **A**. **C** qRT-PCR showing the expressions of the target genes which were downregulated by DRAK1 overexpression. **D** qRT-PCR showing the expressions of the target genes which were upregulated by DRAK1 overexpression. Data in **C** and **D** are representative of three independent experiments. *p < 0.05; **p < 0.01; ***p < 0.001.

Considering that overexpression of DRAK1 suppresses TRAF6-mediated NF-κB signaling, thereby attenuating cell growth and tumorigenesis of HeLa/PTX cells, we investigated whether DRAK1 alters the expression of genes involved in tumor progression. We initially performed transcriptome analysis using RNA sequencing of DRAK1-overexpressing HeLa/PTX cells. Notably, DRAK1 overexpression differentially regulated gene expression in comparison with that in HeLa/PTX cells (Fig. 4A). To gain further insights into the differentially expressed genes associated with DRAK1 overexpression in HeLa/PTX cells, Gene Ontology (GO) and Kyoto Encyclopedia of Genes and Genomes (KEGG) pathway analyses were performed using the DAVID functional annotation tool. The KEGG pathway revealed that DRAK1 altered the expression of genes involved in the cell cycle, cellular senescence, and p53 signaling pathways. Likewise, the GO analysis identified DRAK1 target genes involved in cell cycle regulation, cell cycle phase transition, intracellular transport and protein metabolic processes (Fig. 4B). To validate the RNA sequencing results, qRT-PCR was performed to measure the expression of representative target genes involved in cell cycle regulation. Interestingly, the expression of *E4F1*, *CCNB2*, *CDK10* and *MYC*, which are involved in cell cycle promotion, was upregulated in HeLa/PTX cells compared to that in parental HeLa cells, whereas their expression was reduced by DRAK1 overexpression. Expression of additional cell cycle promoting target genes, *CCNE2, CINP, CDC25L* and *GSTP* showed a similar tendency as expected (Fig. 4C). Conversely, genes associated with cell cycle arrest, such as *RBP5* and *BCCIP*, were induced by DRAK1 overexpression, but suppressed in HeLa/PTX cells (Fig. 4D. Taken together, these findings suggest that DRAK1 inhibits the tumor progression of paclitaxel-resistant cells through the regulation of cell cycle-related target gene expression.

### DRAK1 protein is degraded by the CUL3/SPOP E3 ubiquitin ligase in paclitaxel-resistant cells

**Fig. 5:**
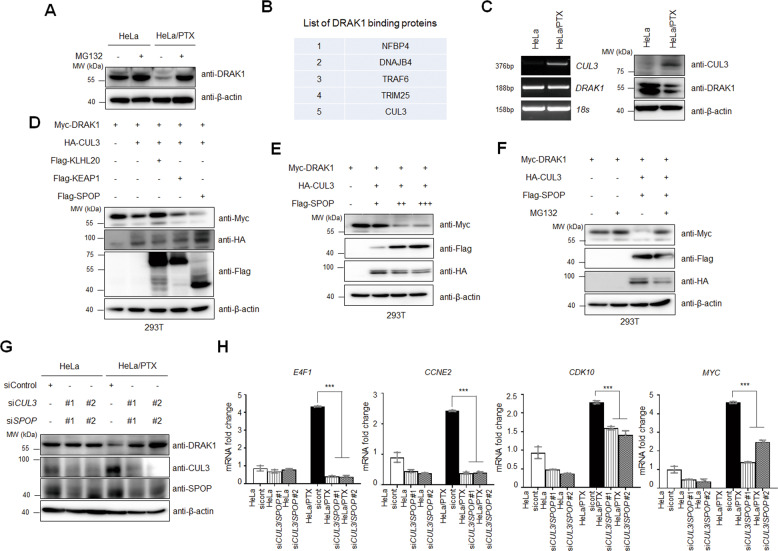
DRAK1 protein is degraded by CUL3/SPOP E3 ubiquitin ligase in paclitaxel-resistant cells. **A** Immunoblot analysis showing DRAK1 expression in HeLa and HeLa/PTX cells with or without MG132 (10μM) treatment for 6 h and β-actin was used for internal control. **B** List of DRAK1 binding protein candidates analyzed from the mass spectrometry-based crosslinking assay. **C** RT-PCR (left) and immunoblot analysis (right) showing CUL3 and DRAK1 expression in HeLa and HeLa/PTX cells. *18* *s* or β-actin was used for internal control, respectively. **D** Immunoblot analysis showing Myc-DRAK1 expression by cotransfection of HA-CUL3 and its adaptor proteins, Flag-KLHL20, Flag-KEAP1, and Flag-SPOP in 293 T cells. **E** Immunoblot analysis showing the expression of Myc-DRAK1 by dose-dependent changes in the expression levels of Flag-SPOP with HA-CUL3. **F** Immunoblot analysis showing Myc-DRAK1 expression by cotransfection of HA-CUL3 and Flag-SPOP with or without MG132 (10 μM) treatment for 4 h. **G** Immunoblot analysis showing endogenous DRAK1 expression in HeLa and HeLa/PTX cells transfected with *CUL3*-siRNAs and *SPOP*-siRNAs. **H** qRT-PCR showing the expressions of downregulated target genes by DRAK1 overexpression validated from Fig. 4C in samples from **G**.

To investigate how DRAK1 protein levels are destabilized in HeLa/PTX cells, we first treated HeLa/PTX cells with the proteasomal degradation inhibitor MG132. DRAK1 expression was rescued by MG132 treatment, suggesting that its protein stability is regulated through the proteasomal degradation pathway (Fig. 5A; Supplementary Fig. S6A). Cross-linking mass spectrometry (MS) analysis was performed to identify DRAK1 interacting proteins involved in DRAK1 protein degradation. Among the promising DRAK1-binding protein candidates by MS analysis, we focused on the CUL3 E3 ubiquitin ligase, which is known to mediate death-associated protein kinase (DAPK) protein degradation [21] (Fig. 5B). First, we confirmed that both *CUL3* mRNA and protein levels were induced in HeLa/PTX cells compared to parental HeLa cells (Fig. 5C; Supplementary Fig. S6B). Considering that CUL3 is a scaffold protein that exploits BTB domain-containing adaptor proteins to recognize substrates, we cotransfected Myc-DRAK1 and HA-CUL3 with various adaptor proteins known to target protein kinases. Notably, CUL3 induced degradation of DRAK1 but cotransfection of CUL3 and SPOP further induced its degradation (Fig. 5D; Supplementary Fig. S6C). Furthermore, DRAK1 expression gradually decreased with increasing the amount of transfected SPOP protein compared to that with CUL3 transfection alone, suggesting that CUL3 more effectively regulated DRAK1 expression with SPOP adaptor protein expression (Fig. 5E; Supplementary Fig. S6D). The reduced expression of DRAK1 in the presence of CUL3 and SPOP was rescued upon MG132 treatment, indicating that DRAK1 protein was degraded through a proteasomal degradation pathway mediated by CUL3 and SPOP (Fig. 5F; Supplementary Fig. S6E). To confirm that reduced endogenous DRAK1 protein levels in HeLa/PTX cells were regulated by CUL3 and SPOP, we transiently transfected parental HeLa and HeLa/PTX cells with *CUL3* and *SPOP* siRNA. As expected, DRAK1 expression in HeLa cells was not altered, however, it was dramatically restored by CUL3 and SPOP-knockdown in HeLa/PTX cells (Fig. 5G; Supplementary Fig. S6F). Given that DRAK1 overexpression suppressed the expression of cell cycle-promoting target genes such as *E4F1*, *CCNE2*, *CDK10*, and *MYC* shown in Fig. 4C, we analyzed the expression of those target genes in CUL3- and SPOP-depleted HeLa and HeLa/PTX cells by qRT-PCR. Indeed, enhanced target gene expression in HeLa/PTX cells was significantly reduced as DRAK1 protein was restored by knockdown of CUL3 and SPOP expressions (Fig. 5H). Collectively, these results suggest that the stability of the DRAK1 protein is reduced by the CUL3 E3 ubiquitin ligase along with the SPOP adaptor protein through the proteasomal degradation pathway.

### DRAK1 is degraded through K48-linked polyubiquitination induced by CUL3/SPOP E3 ubiquitin ligase

**Fig. 6:**
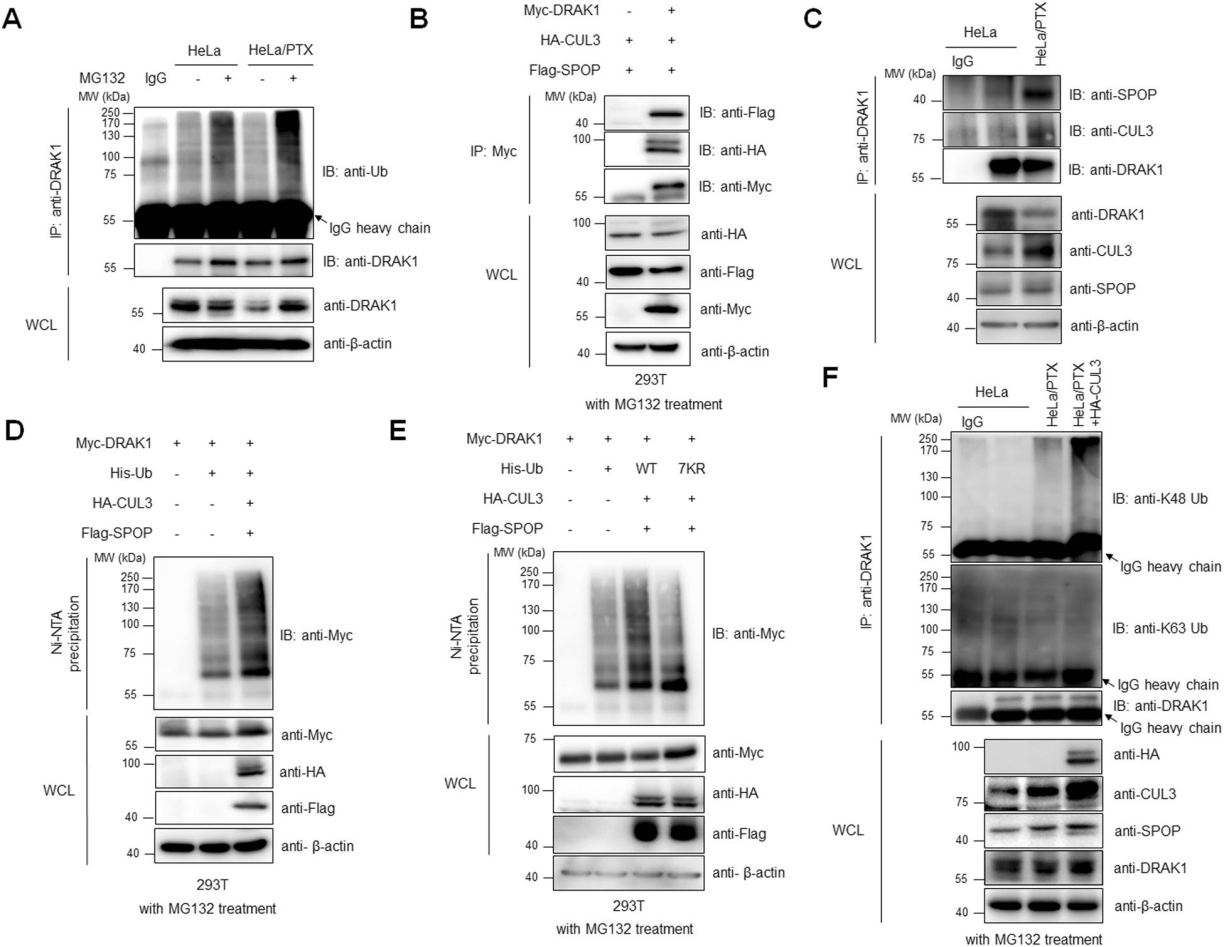
DRAK1 is degraded through K48-linked polyubiquitination induced by CUL3/SPOP E3 ubiquitin ligase. **A** Immunoprecipitation assay showing endogenous DRAK1 ubiquitination in HeLa and HeLa/PTX cells upon MG132 (10μM) treatment for 6 h. **B** Immunoprecipitation assay showing the interaction of Myc-DRAK1 with HA-CUL3 and Flag-SPOP in 293 T cells. **C** Immunoprecipitation assay showing the endogenous interaction of DRAK1 with CUL3 and SPOP in HeLa and HeLa/PTX cells. **D** In vivo ubiquitination assay showing Myc-DRAK1 ubiquitination by cotransfection of HA-CUL3 and Flag-SPOP in 293 T cells with MG132 (10 μM) treatment for 6 h. **E** In vivo ubiquitination assay showing Myc-DRAK1 ubiquitination by cotransfection of HA-CUL3 and Flag-SPOP in 293 T cells using Ni-NTA pull down of His-Ubiquitin WT or His-Ubiquitin 7KR mutant (mutating all seven lysines to arginines) with MG132 (10μM) treatment for 6 h. **F** Immunoprecipitation assay showing endogenous DRAK1 ubiquitin chain linkages. HA-CUL3 ectopically transfected in HeLa/PTX and indicated samples were immunoprecipitated with anti-DRAK1 antibody, followed by immunoblotting with anti-K48 Ub (K48-linkage specific Ubiquitin) or anti-K63 Ub (K63-linkage specific Ubiquitin) antibodies upon MG132 (10 μM) treatment for 6 h.

Considering that CUL3 ubiquitin ligase induces ubiquitination of its substrate to promote the proteasomal degradation pathway, we initially performed an in vivo ubiquitination assay to detect endogenous DRAK1 ubiquitination upon MG132 treatment. Interestingly, endogenous ubiquitination of DRAK1 was enhanced in MG132-treated HeLa/PTX cells compared to that in HeLa cells (Fig. 6A). To determine whether the induction of DRAK1 ubiquitination was directly mediated through interaction with the CUL3/SPOP E3 ubiquitin ligase, 293 T cells were transiently transfected with Myc-DRAK1, HA-CUL3, and Flag-SPOP plasmids. Indeed, immunoprecipitation assays showed that DRAK1 interacted with both CUL3 and SPOP proteins (Fig. 6B). Furthermore, we confirmed that the endogenous interaction between DRAK1 and CUL3/SPOP was increased in HeLa/PTX cells compared to that in HeLa cells (Fig. 6C). Our findings led us to examine whether DRAK1 is ubiquitinated by the CUL3/SPOP E3 ubiquitin ligase through an in vivo ubiquitination assay in 293 T cells. Intriguingly, the polyubiquitin chain of DRAK1 was enhanced in the presence of CUL3 and SPOP proteins, and there was a notable decrease in the amount of ubiquitin chain of DRAK1 with cotransfection of His-Ub 7KR (mutating all seven lysines to arginines, thus blocking Ub chain formation), suggesting that polyubiquitination of DRAK1 was induced by CUL3 and SPOP E3 ligases (Fig. 6D, E). Having demonstrated that CUL3/SPOP is a DRAK1-interacting E3 ligase that destabilizes DRAK1 by inducing polyubiquitination, we further characterized a specific type of polyubiquitin chains of DRAK1 by CUL3/SPOP E3 ligases. Interestingly, immunoprecipitation assay showed that ectopic expression of HA-CUL3 enhanced the endogenous K48-linked polyubiquitin chains of DRAK1 in HeLa/PTX cells, whereas no significant induction of K63-linked polyubiquitination of endogenous DRAK1 was observed (Fig. 6F). Taken together, these findings suggest that DRAK1 is destabilized through the K48-linked polyubiquitin-mediated proteasomal degradation pathway promoted by the CUL3-SPOP E3 ubiquitin ligase in paclitaxel-resistant cells.

## Discussion

Cancer cells can acquire resistance to anticancer drugs when the expression levels of their target proteins change after long-term treatment. Here, we report that DRAK1 protein was specifically degraded through K48-linked polyubiquitination induced by CUL3/SPOP E3 ubiquitin ligase in paclitaxel-resistant cervical cancer cells. DRAK1 destabilization resulted in TRAF6 upregulation and TRAF6-mediated NF-κB activation, thereby promoting tumorigenesis of paclitaxel-resistant cells. Ectopic overexpression of DRAK1 in paclitaxel-resistant cells reduced tumor progression, along with induction of G0/G1 arrest and downregulation of cell cycle promoting target genes.

Paclitaxel is widely used for the treatment of various cancers including cervical, breast, ovarian, brain, prostate, and lung cancers. The combination of paclitaxel with the various other anticancer agents is an approved regimen for the treatment of recurrent and advanced cancers [22–26]. Considering that paclitaxel has been more efficient in combination with the other anticancer agents in recent clinical trials, further comprehensive work is needed to determine the function of DRAK1 associated with other anticancer agents.

Previous studies have demonstrated the oncogenic role of TRAF6 in various cancers and the upregulation of TRAF6/NF-κB axis conferred chemoresistance of cancer cells [27, 28], however, the function of TRAF6 in association with paclitaxel resistance in cervical cancer has remained unclear. In this study, we investigated that a decreased DRAK1 protein stability followed by upregulation of TRAF6 levels, and TRAF6-mediated NF-κB signaling pathway, promotes the malignancy of paclitaxel-resistant cervical cancer cells. In view of our previous observation that blockade of ubiquitination on TRAF6 by DRAK1 was a key mechanism regulating TRAF6 stability in cervical cancer cells [12], targeting TRAF6 E3 ligase activity associated with drug resistance in other cancers deserve further investigation.

Our study also showed that DRAK1 acts as a tumor suppressor in paclitaxel-resistant cervical cancer cells. Forced expression of DRAK1 in cervical cancer cells with acquired resistance to paclitaxel decreased cell growth and tumorigenesis of paclitaxel-resistant cells as well as cellular resistance to paclitaxel by downregulating TRAF6 expression and NF-κB activation. We elucidated the role of DRAK1 as a tumor suppressor in cervical cancer and recent studies showed that induction of DRAK1 expression reduced the cell growth in testicular and prostrated cancer cells [29, 30], whereas other reports suggest that DRAK1 promotes tumorigenic potential in head and neck cancer and glioblastoma cells [13, 31, 32]. With respect to many aspects of this protein, including the importance of its expression, target substrates, and binding partners, further investigation will be needed to address the distinct role of DRAK1 in drug resistance in other tissues.

Our findings raise questions of which of the target genes regulated by DRAK1 are involved in phenotypic changes in HeLa/PTX cells. In this study, based on RNA sequencing analysis of DRAK1-overexpressing HeLa/PTX, HeLa/PTX, and parental HeLa cells, we found that target genes involved in cell cycle regulation were mostly altered by changes in DRAK1 expression. Among the target genes downregulated by DRAK1 overexpression, *MYC* has been reported to have sustained induction by paclitaxel leading to tumor progression of PDAC [33, 34]. Given that transactivation of *MYC* through the NF-κB pathway induces cell proliferation and cell cycle promotion in a variety of cancer cells [35], our observations may provide a mechanistic explanation to overcome paclitaxel resistance.

In this study, we also elucidated the detailed mechanisms by which DRAK1 protein is negatively regulated in paclitaxel-resistant cells. Previously, it was reported that the *DRAK1* transcript level was directly upregulated by p53 bound to an upstream element of the *DRAK1* gene and that DRAK1 knockdown resulted in cisplatin resistance in human embryonal carcinoma cells [29]; however, it had not been determined whether the DRAK1 protein levels were regulated through posttranslational modifications. In our study, we revealed that the DRAK1 protein was degraded through K48-linked polyubiquitination catalyzed by the CUL3/SPOP E3 ubiquitin ligase in HeLa/PTX cells without altering its mRNA expression. Although extensive studies have demonstrated that CUL3 ubiquitin ligase is involved in diverse cellular processes regulating tumorigenesis and that inhibition of CUL3 sensitizes ovarian cancer cells to cisplatin [36], the precise mechanism by which CUL3 is involved in cellular resistance in cervical cancer has yet to be elucidated. Our results showed that upregulation of CUL3 expression resulted in the degradation of DRAK1 protein, which consequently increased TRAF6 levels and TRAF6-mediated NF-κB signaling cascades in HeLa/PTX cells.

In conclusion, we demonstrated that the DRAK1 protein is destabilized by the CUL3/SPOP E3 ubiquitin ligase through a K48-linked polyubiquitin-dependent proteasomal degradation pathway in paclitaxel-resistant cells. In addition, given that DRAK1 expression is significantly associated with paclitaxel resistance in cervical cancer cells and tumorigenesis of paclitaxel-resistant cells, DRAK1 may serve as a potential predictive value for overcoming the therapeutic resistance of cervical cancers.

## Material and Methods

### Cell culture and reagents

The human cervical cancer cell lines HeLa, CaSki, and 293 T were obtained from the American Type Culture Collection (ATCC). These cell lines were cultured at 37 °C in RPMI1640 medium with 10% fetal bovine serum (FBS) and 1% penicillin/streptomycin. Medium and reagents for cell culture were purchased from WELGENE, Inc., Republic of Korea. Paclitaxel-resistant sublines from HeLa cells were generated by stepwise increased in paclitaxel concentrations from 1 nM to 50 nM over 3 months. The resulting paclitaxel-resistant HeLa cells, designated as HeLa/PTX, were maintained with 50 nM paclitaxel in the cell culture medium. DNA and RNAi transfections were carried out using Polyethylenimine (PEI, Sigma-Aldrich), Fugene® HD (promega) or Lipofectamine® RNAiMAX Reagent (Invitrogen), respectively, according to the manufacturer’s instructions. Paclitaxel (T7402) and MG132 (C2211) were purchased from Sigma-Aldrich.

### Plasmids

Full-length human CUL3 and SPOP complementary DNA (cDNA) was amplified from HeLa cDNA by PCR and subcloned into the EcoRI and XhoI sites or XhoI and NheI of the pCS4-3HA vector or pCS4-3Flag vector (Addgene), resulting in HA-CUL3 and Flag-SPOP. Human KLHL20, KEAP1 cDNA was amplified from HeLa cDNA by PCR and subcloned into the XhoI and NheI of the pCS4-3Flag vector (Addgene), resulting in Flag-KLHL20 and Flag-KEAP1. Human HA-Ub and HA-Ub mutants (7KR) were kindly provided by S.H.P. (Sungkyunkwan University, Korea). Myc-DRAK1 and LPCX-Myc-DRAK1 plasmids were generated as described in our previous study [12]. Primer sequences for PCR amplification in this study are listed in Supplementary Table 1.

### Generation of stable cell lines

For generation of retroviruses, GP2-293 cells were plated on a 100-mm culture plate 24 h before transfection. Transfection was performed using polyethylenimine (PEI) with 10 μg DNA (LPCX-Myc-DRAK1 or LPCX control) and 5 μg VSV-G per plate. After transfection, the conditioned medium containing recombinant retroviruses was collected and filtered through 0.45-μm sterilization filters. Then, 3 ml of filtered retroviruses was applied to HeLa and HeLa/PTX cells, which had been plated for 18 h before infection in a 100-mm culture dish. Polybrene (Sigma-Aldrich) was added to a final concentration of 8 μg/ml, and the supernatants were incubated with the cells for 8 h. The medium was aspirated and replaced with fresh viral supernatant, and the procedure was repeated. After infection, the cells were placed in fresh growth medium for 24 h and cultured as usual. Selection with 2 μg/ml puromycin (Sigma-Aldrich) was initiated 48 h after infection. For transient knockdown of *CUL3* and *SPOP*, 40 μM of siCUL3 or SPOP RNA duplex (Bioneer) was transfected using Lipofectamine® RNAiMAX Reagent (Invitrogen) in target cell lines. Target sequences of siRNAs in this study are listed in Supplementary Table 1.

### Luciferase assay

NF-κB promoter luciferase was transfected into each cell lines using FuGENE HD (Promega). The luciferase activities were analyzed using the Luciferase Assay System Kit (Promega) according to the manufacturer’s protocol. All assays were performed in triplicate, and the luciferase activities were normalized against β-galactosidase activities.

### RNA extraction, RT-PCR, and quantitative real-time PCR (qRT-PCR)

Total RNA from each cell lines was extracted using the easy-BLUE Total RNA extraction kit (promega), according to the protocol provided by the manufacturer. The cDNA was synthesized by reverse transcription (RT) with 2 μg of purified RNA using M-MLV reverse transcriptase (promega, M1705). The synthesized cDNA was amplified by PCR using specific primers. PCR products were visualized by electrophoresis on 1.5% agarose gels with Redsafe (Chembio, 21141) staining and analyzed with an ImageQuant LAS 4000 image analyzer (GE Healthcare). The 18 S rRNA gene was used as an internal control. Quantitative real-time PCR (qRT-PCR) was performed with the proper primers using 2X SYBR Green PCR Master Mix (Takara) and conducted by Quant Studio 5 (Applied Biosystems). All reactions were performed at least three times independently. Primer sequences for PCR amplification in this study are listed in Supplementary Table 1.

### Immunoblot analysis and immunoprecipitation assay

Cells were washed twice in cold PBS and lysed in IP buffer (50 mM Tris, pH 7.4, 150 mM NaCl, 1% Triton X-100, 0.5% sodium deoxycholate, 2 mM EDTA, and 10% glycerol) plus phosphatase and protease inhibitors (Roche). Proteins from whole-cell extracts were quantified using BCA solution and boiled samples with 5X sample loading buffer at 95 °C. For immunoprecipitation assay, plasmids were transfected using PEI in 293 T cells and after 36 h cells were harvested and lysed in IP buffer. Whole-cell extracts were incubated with the appropriate primary antibodies overnight at 4 °C. Antibody-bound proteins were precipitated with Dynabeads (Thermo Fisher Scientific) according to the manufacturer’s protocol. The beads were washed three times with lysis buffer and then eluted in 2X SDS sample loading buffer. Eluted proteins were separated by SDS–polyacrylamide gel electrophoresis, transferred to PVDF membranes (Millipore), and detected using appropriate primary antibodies coupled with a horseradish peroxidase-conjugated secondary antibody by chemiluminescence (GE Healthcare). The primary antibodies used are listed in Supplemental Table 2.

### In vivo ubiquitination assay

Cells suspended in TBS buffer (10 mM Tris-HCl (pH 8.0), 150 mM NaCl, 1% SDS) were added to ice-cold TBS containing 2% SDS buffer. The cell mixture was vortexed for 5 s, following which 5 mM NEM, a protease inhibitor, was added. The lysates were boiled for 10 min and diluted 10-fold with dilution buffer (TBS buffer, 1% Triton X-100, 5 mM NEM). The protein lysates were incubated with the indicated antibodies while rotating overnight at 4 °C. Antibody-bound proteins were precipitated with Dynabeads (Thermo Fisher Scientific) for 1 h. The precipitates were washed once with washing buffer A (TBS buffer, 1% Triton X-100, 0.1% SDS) and twice with washing buffer B (TBS buffer, 1% Triton X-100). The proteins were eluted in 2× SDS sample loading buffer, boiled for 5 min and subjected to immunoblot analysis.

### Ni-NTA ubiquitination assay

Cells were suspended in cold PBS containing 5 mM NEM. The cells were then lysed in binding buffer (6 M Guanidine HCl, 0.1 M Na_2_HPO_4_ and NaH_2_PO_4_, 0.01 M Tris-HCl (pH 8.0), 10 mM β-mercaptoethanol, 5 mM NEM, 20 mM imidazole), and the lysates were added to Ni-NTA agarose beads (Qiagen) and rotated overnight at 4 °C. The precipitates were washed once with washing buffer A (6 M Guanidine HCl, 0.1 M Na_2_HPO_4_ and NaH_2_PO_4_, 0.01 M Tris-HCl (pH 8.0), 10 mM β-mercaptoethanol) by rotating at RT for 5 min. The precipitates were washed once with buffer B (8 M Urea, 0.1 M Na_2_HPO_4_ and NaH_2_PO_4,_ 0.01 M Tris-HCl (pH 8.0), 10 mM β-mercaptoethanol), buffer C (8 M Urea, 0.1 M Na_2_HPO_4_ and NaH_2_PO_4,_ 0.01 M Tris-HCl (pH 6.3), 10 mM β-mercaptoethanol, 0.2% Triton X-100) and buffer D (8 M Urea, 0.1 M Na_2_HPO_4_ and NaH_2_PO_4,_ 0.01 M Tris-HCl (pH 6.3), 10 mM β-mercaptoethanol, 0.1% Triton X-100). The proteins were eluted in elution buffer (2× SDS sample loading buffer, 0.72 M β-mercaptoethanol, 250 mM imidazole) and subjected to immunoblot analysis.

### Cell viability assay

2 × 10^3^ of each cell lines were seeded in 96-well plates 24 h prior to paclitaxel treatment. Paclitaxel was diluted to a range of concentrations (2.5-40 nM) in RPMI1640 culture medium and was added to each well; cells were incubated for 48–72 h. MTT (M5655, Sigma-Aldrich) stock solution (5 mg/ml) was added to each culture well being assayed to equal one-tenth the original culture volume and incubated for 3 to 4 h. Cell medium was removed and attached cells and the converted dye was solubilized with DMSO. Absorbance of converted dye was measured at a wavelength of 570 nm with background subtraction at 630 nm.

### Tumor spheroid formation assay

1 × 10^3^ of each cell lines were resuspended in RPMI1640 medium with 10% FBS, 0.2 μg/mL EGF (236-EG-200, R&D system), 0.1 μg/mL bFGF (100-18B, PeproTech), B27 (17504, Invitrogen) and 4 μg/mL Insulin (12585-014, GIBCO) and seeded in ultra-low attachment 6-well plates (3471, Corning). 10 nM of paclitaxel was treated every 2 days. After 10 days, the spheroids were observed by microscope. The spheroid areas were measured by image J software and calculated using *A* = 2πrR (*r*=shortest diameter, *R* = longest diameter).

### In vivo tumor-formation assay

All procedures were approved by the Woojung Bio Animal facility (Suwon, Korea) and Medpacto, Inc. Animal facility (Seoul, Korea). Animal protocols were performed in compliance with the relevant ethical standards. For in vivo tumor-formation assay, a total of 5 ×10^6^ parental HeLa, paclitaxel-resistant HeLa cells, and DRAK1-overexpressing paclitaxel-resistant HeLa cells were suspended in 1:3 PBS/hydrogel (TheWell Bioscience) solution and subcutaneously injected into 6-week-old female NOD/ShiLtJ-Rag2em1AMC Il2rgem1AMC (NRGA, Joong Ah Bio) mice (*n* = 8 per group) to measure tumor growth. Samples sizes were determined based on the current research methods for xenograft models. Tumor size was monitored weekly started from 4 weeks after injection and tumor volume was calculated using the formula *V* = (A x B^2^)/2, where *V* is volume (mm^3^), A is long diameter (mm), and B is short diameter (mm). No randomization was used for animal studies and blinding was not needed to perform the animal studies. Tumor tissues were fixed in formaldehyde solution and were embedded in paraffin block. The slide section was stained with indicated antibody for immunohistochemistry. The primary antibodies used are listed in Supplementary Table 2.

### Human cervical cancer tissue microarray and immunohistochemistry

Human cervical cancer tissues from the surgical section at Seoul National University College of Medicine (Seoul, Korea) were collected with approval by the institutional review board (IRB approval number 1707-063-869). All procedures involving human participants were performed in compliance with the relevant ethical standards. Human participants consented for publication. For immunohistochemistry, TMA sections were fixed and stained using anti-DRAK1 antibody (Origene, am20971PU-N) and anti-TRAF6 antibody (Santa Cruz, sc-8409) and counterstained with hematoxylin. After staining, slides were scored under a microscope and analyzed. Analysis of tissue microarray was performed in by blinded pathologist.

### RNA sequencing

The total RNAs were treated with DNase I, purified with miRNeasy Mini Kit (Qiagen) and subsequently quality checked using an Agilent 2100 Bioanalyzer (Agilent). An Illumina platform (Illumina) was used to analyze transcriptomes with 90 bp paired-end library. Samples were pair-end sequenced with the Illumina HiSeq 2500 sequencing platform. We filtered low quality reads using Cutadapt v2.8 [37] with quality cutoff 20 and minimum length 50. Filtered reads were mapped to the GRCh38 genome using STAR v2.7.1 [38]. Gene expression read counts were quantified using RSEM v1.3.1 [39] and normalized by the reads per kilobase of transcript per million fragments mapped (RPKM). The differentially expressed genes in Fig. 4A were obtained using edgeR v3.28.1 [40] by comparing HeLa LPCX versus HeLa/PTX LPCX and Myc-DRAK1 HeLa/PTX versus HeLa/PTX LPCX with corrected *p*-value < 0.05 and absolute log2 fold change > 1.

### GO, KEGG pathway analysis

The enriched GO and KEGG pathway terms for Fig. 4B were obtained from Enrichr software [41].

### Cell cycle analysis

A total of 1 × 10^4^ cells was trypsinized and harvested. Fix the cells in cold 70% ethanol by adding dropwise to the pellet with mild voltexing for overnight at -20 °C. Centrifuged at 2000rpm for 5 minutes and discard the supernatant. Resuspend the fixed cells in PBS and centrifuged at 2000rmp for 5 minutes for two rounds of washes. To ensure that only DNA is stained, treat cells with 50 μl of 100 μg/ml Ribonuclease A (Sigma Aldrich, 10109169001). Add 425 μl of Cell staining buffer (BioLegend, 420201) and 25 μl of Propidium Iodide solution (BioLegend, 421301). Cell cycle distribution was analyzed using BD FACSCanto II flow cytometer and FCS Express 7 software.

### Cross-linking for protein interaction analysis

Crosslinking for mass spectrometry analysis was performed as described previously [42]. In particular, cells were fixed in 1% formaldehyde in PBS. Eluted proteins were loaded on SDS-polyacrylamide gel and separated by electrophoresis. Crosslinked proteins on gel were stained in Coomassie blue (Tech & Innovation) and analyzed through mass spectrometry at Korea Basic Science Institute (Daejeon, Korea).

### Statistical analysis

All experiments were repeated at least in triplicate and the results presented were the mean of three replicates. Statistical significance was calculated by using GraphPad Prism 5 software. For comparison between sample groups, the unpaired two-tailed Student’s *t* test was used. *P* value less than 0.05 was considered statistically significant. Error bars represents the mean ± S.D. of each independent experiment.

## Supplementary information

Supplementary Figure S1

Supplementary Figure S2

Supplementary Figure S3

Supplementary Figure S4

Supplementary Figure S5

Supplementary Figure S6

Supplementary Figure Legends

Supplementary Table 1

Supplementary Table 2

## Data Availability

The datasets generated during and/or analyzed during the current study are available from the corresponding author on reasonable request.

## References

[1] Kudelka AP, Winn R, Edwards CL, Downey G, Greenberg H, Dakhil SR (1996). Activity of paclitaxel in advanced or recurrent squamous cell cancer of the cervix. Clin Cancer Res.

[2] Pectasides D, Kamposioras K, Papaxoinis G, Pectasides E (2008). Chemotherapy for recurrent cervical cancer. Cancer Treat Rev.

[3] Mansoori B, Mohammadi A, Davudian S, Shirjang S, Baradaran B (2017). The Different Mechanisms of Cancer Drug Resistance: A Brief Review. Adv Pharm Bull.

[4] Cree IA, Charlton P (2017). Molecular chess? Hallmarks of anti-cancer drug resistance. BMC Cancer.

[5] Vasan N, Baselga J, Hyman DM (2019). A view on drug resistance in cancer. Nature..

[6] Mabuchi S, Ohmichi M, Nishio Y, Hayasaka T, Kimura A, Ohta T (2004). Inhibition of inhibitor of nuclear factor-kappaB phosphorylation increases the efficacy of paclitaxel in in vitro and in vivo ovarian cancer models. Clin Cancer Res.

[7] Fraser M, Leung B, Jahani-Asl A, Yan X, Thompson WE, Tsang BK (2003). Chemoresistance in human ovarian cancer: the role of apoptotic regulators. Reprod Biol Endocrinol.

[8] Nakanishi C, Toi M (2005). Nuclear factor-kappaB inhibitors as sensitizers to anticancer drugs. Nat Rev Cancer.

[9] Fang J, Muto T, Kleppe M, Bolanos LC, Hueneman KM, Walker CS (2018). TRAF6 Mediates Basal Activation of NF-kappaB Necessary for Hematopoietic Stem Cell Homeostasis. Cell Rep..

[10] Shi JH, Sun SC (2018). Tumor Necrosis Factor Receptor-Associated Factor Regulation of Nuclear Factor kappaB and Mitogen-Activated Protein Kinase Pathways. Front Immunol.

[11] Starczynowski DT, Lockwood WW, Delehouzee S, Chari R, Wegrzyn J, Fuller M (2011). TRAF6 is an amplified oncogene bridging the RAS and NF-kappaB pathways in human lung cancer. J Clin Invest.

[12] Park Y, Pang K, Park J, Hong E, Lee J, Ooshima A (2020). Destablilization of TRAF6 by DRAK1 Suppresses Tumor Growth and Metastasis in Cervical Cancer Cells. Cancer Res.

[13] Park Y, Kim W, Lee JM, Park J, Cho JK, Pang K (2015). Cytoplasmic DRAK1 overexpressed in head and neck cancers inhibits TGF-beta1 tumor suppressor activity by binding to Smad3 to interrupt its complex formation with Smad4. Oncogene..

[14] Wittig R, Nessling M, Will RD, Mollenhauer J, Salowsky R, Munstermann E (2002). Candidate genes for cross-resistance against DNA-damaging drugs. Cancer Res.

[15] Guo Q, Chen Y, Wu Y (2009). Enhancing apoptosis and overcoming resistance of gemcitabine in pancreatic cancer with bortezomib: a role of death-associated protein kinase-related apoptosis-inducing protein kinase 1. Tumori..

[16] Gao J, Liu D, Li J, Song Q, Wang Q (2016). Effect of STK17A on the sensitivity of ovarian cancer cells to paclitaxel and carboplatin. Oncol Lett.

[17] Tang H, Liu YJ, Liu M, Li X (2007). Establishment and gene analysis of an oxaliplatin-resistant colon cancer cell line THC8307/L-OHP. Anticancer Drugs.

[18] Chen RH (2020). Cullin 3 and Its Role in Tumorigenesis. Adv Exp Med Biol.

[19] Wang L, Lin M, Chu M, Liu Y, Ma J, He Y (2020). SPOP promotes ubiquitination and degradation of LATS1 to enhance kidney cancer progression. EBioMedicine.

[20] Nunes AS, Barros AS, Costa EC, Moreira AF, Correia IJ (2019). 3D tumor spheroids as in vitro models to mimic in vivo human solid tumors resistance to therapeutic drugs. Biotechnol Bioeng.

[21] Lee YR, Yuan WC, Ho HC, Chen CH, Shih HM, Chen RH (2010). The Cullin 3 substrate adaptor KLHL20 mediates DAPK ubiquitination to control interferon responses. EMBO J.

[22] Abu Samaan TM, Samec M, Liskova A, Kubatka P, Busselberg D (2019). Paclitaxel’s Mechanistic and Clinical Effects on Breast Cancer. Biomolecules.

[23] Kampan NC, Madondo MT, McNally OM, Quinn M, Plebanski M (2015). Paclitaxel and Its Evolving Role in the Management of Ovarian Cancer. Biomed Res Int.

[24] Machida H, Moeini A, Ciccone MA, Mostofizadeh S, Takiuchi T, Brunette LL (2018). Efficacy of Modified Dose-dense Paclitaxel in Recurrent Cervical Cancer. Am J Clin Oncol.

[25] Li CM, Lu Y, Chen J, Costello TA, Narayanan R, Dalton MN (2012). Orally bioavailable tubulin antagonists for paclitaxel-refractory cancer. Pharm Res.

[26] Ramalingam S, Belani CP (2004). Paclitaxel for non-small cell lung cancer. Expert Opin Pharmacother.

[27] Meng Q, Liang C, Hua J, Zhang B, Liu J, Zhang Y (2020). A miR-146a-5p/TRAF6/NF-kB p65 axis regulates pancreatic cancer chemoresistance: functional validation and clinical significance. Theranostics.

[28] Xie C, Zhang LZ, Chen ZL, Zhong WJ, Fang JH, Zhu Y (2020). A hMTR4-PDIA3P1-miR-125/124-TRAF6 Regulatory Axis and Its Function in NF kappa B Signaling and Chemoresistance. Hepatology.

[29] Mao P, Hever MP, Niemaszyk LM, Haghkerdar JM, Yanco EG, Desai D (2011). Serine/threonine kinase 17A is a novel p53 target gene and modulator of cisplatin toxicity and reactive oxygen species in testicular cancer cells. J Biol Chem.

[30] Manivannan P, Reddy V, Mukherjee S, Clark KN, Malathi K (2019). RNase L Induces Expression of A Novel Serine/Threonine Protein Kinase, DRAK1, to Promote Apoptosis. Int J Mol Sci.

[31] Mao P, Hever-Jardine MP, Rahme GJ, Yang E, Tam J, Kodali A (2013). Serine/threonine kinase 17A is a novel candidate for therapeutic targeting in glioblastoma. PLoS One.

[32] Chen AS, Wardwell-Ozgo J, Shah NN, Wright D, Appin CL, Vigneswaran K (2019). Drak/STK17A Drives Neoplastic Glial Proliferation through Modulation of MRLC Signaling. Cancer Res.

[33] Bourgarel-Rey V, El Khyari S, Rimet O, Bordas B, Guigal N, Braguer D (2000). Opposite effects of antimicrotubule agents on c-myc oncogene expression depending on the cell lines used. Eur J Cancer.

[34] Parasido E, Avetian GS, Naeem A, Graham G, Pishvaian M, Glasgow E (2019). The Sustained Induction of c-MYC Drives Nab-Paclitaxel Resistance in Primary Pancreatic Ductal Carcinoma Cells. Mol Cancer Res.

[35] Bourgarel-Rey V, Vallee S, Rimet O, Champion S, Braguer D, Desobry A (2001). Involvement of nuclear factor kappaB in c-Myc induction by tubulin polymerization inhibitors. Mol Pharm.

[36] Jazaeri AA, Shibata E, Park J, Bryant JL, Conaway MR, Modesitt SC (2013). Overcoming platinum resistance in preclinical models of ovarian cancer using the neddylation inhibitor MLN4924. Mol Cancer Ther.

[37] Robinson MD, McCarthy DJ, Smyth GK (2010). edgeR: a Bioconductor package for differential expression analysis of digital gene expression data. Bioinformatics.

[38] Dobin A, Davis CA, Schlesinger F, Drenkow J, Zaleski C, Jha S (2013). STAR: ultrafast universal RNA-seq aligner. Bioinformatics.

[39] Li B, Dewey CN (2011). RSEM: accurate transcript quantification from RNA-Seq data with or without a reference genome. BMC Bioinforma.

[40] Carpeggiani C, Landi P, Michelassi C, Barberini E, L’Abbate A (2011). Long-term prognosis in stable angina; medical treatment or coronary revascularization in patients younger than 70 years?. Int J Cardiol.

[41] Kuleshov MV, Jones MR, Rouillard AD, Fernandez NF, Duan Q, Wang Z (2016). Enrichr: a comprehensive gene set enrichment analysis web server 2016 update. Nucleic Acids Res.

[42] Pang K, Park J, Ahn SG, Lee J, Park Y, Ooshima A (2019). RNF208, an estrogen-inducible E3 ligase, targets soluble Vimentin to suppress metastasis in triple-negative breast cancers. Nat Commun.

