# Supplementary figures and images for: Degradation of DRAK1 by CUL3/SPOP E3 Ubiquitin ligase promotes tumor growth of paclitaxel-resistant cervical cancer cells
Source: Cell Death Dis. 2022 Feb 22;13(2):169. doi: 10.1038/s41419-022-04619-w (PMC8863983; doi:10.1038/s41419-022-04619-w)

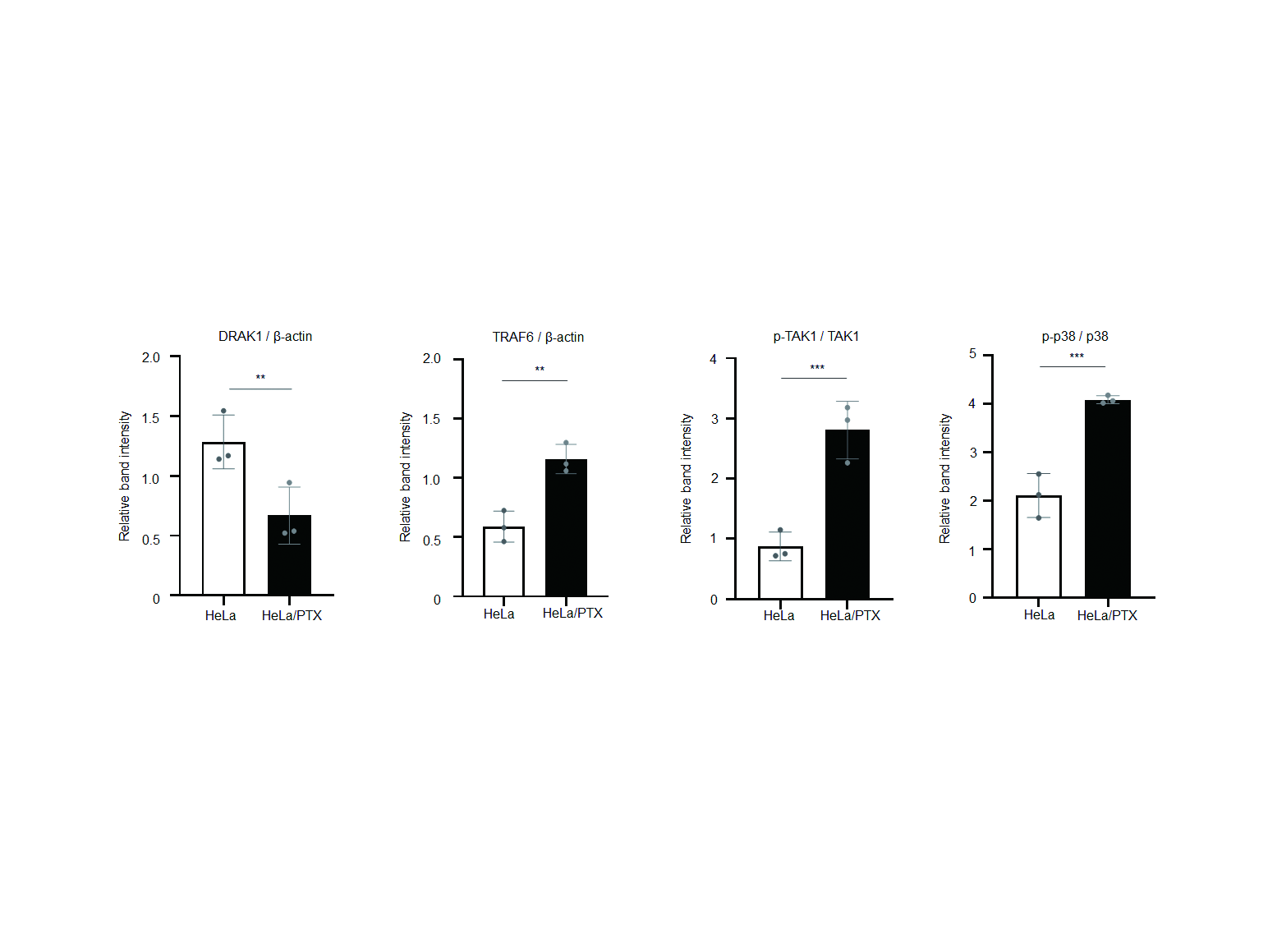

Supplement: Supplementary file 1 — Supplementary Figure S1 [file 41419_2022_4619_MOESM1_ESM.tif]

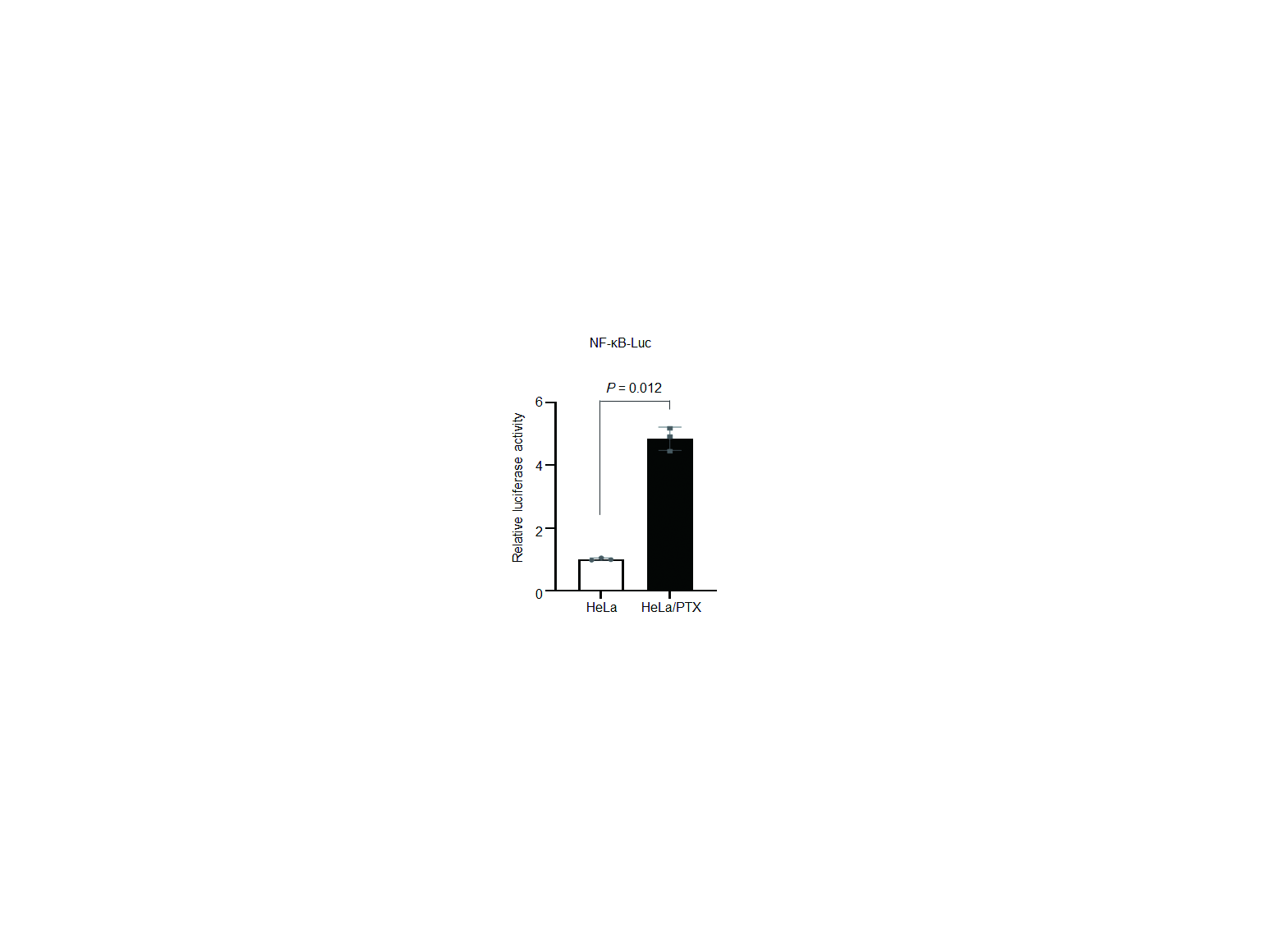

Supplement: Supplementary file 2 — Supplementary Figure S2 [file 41419_2022_4619_MOESM2_ESM.tif]

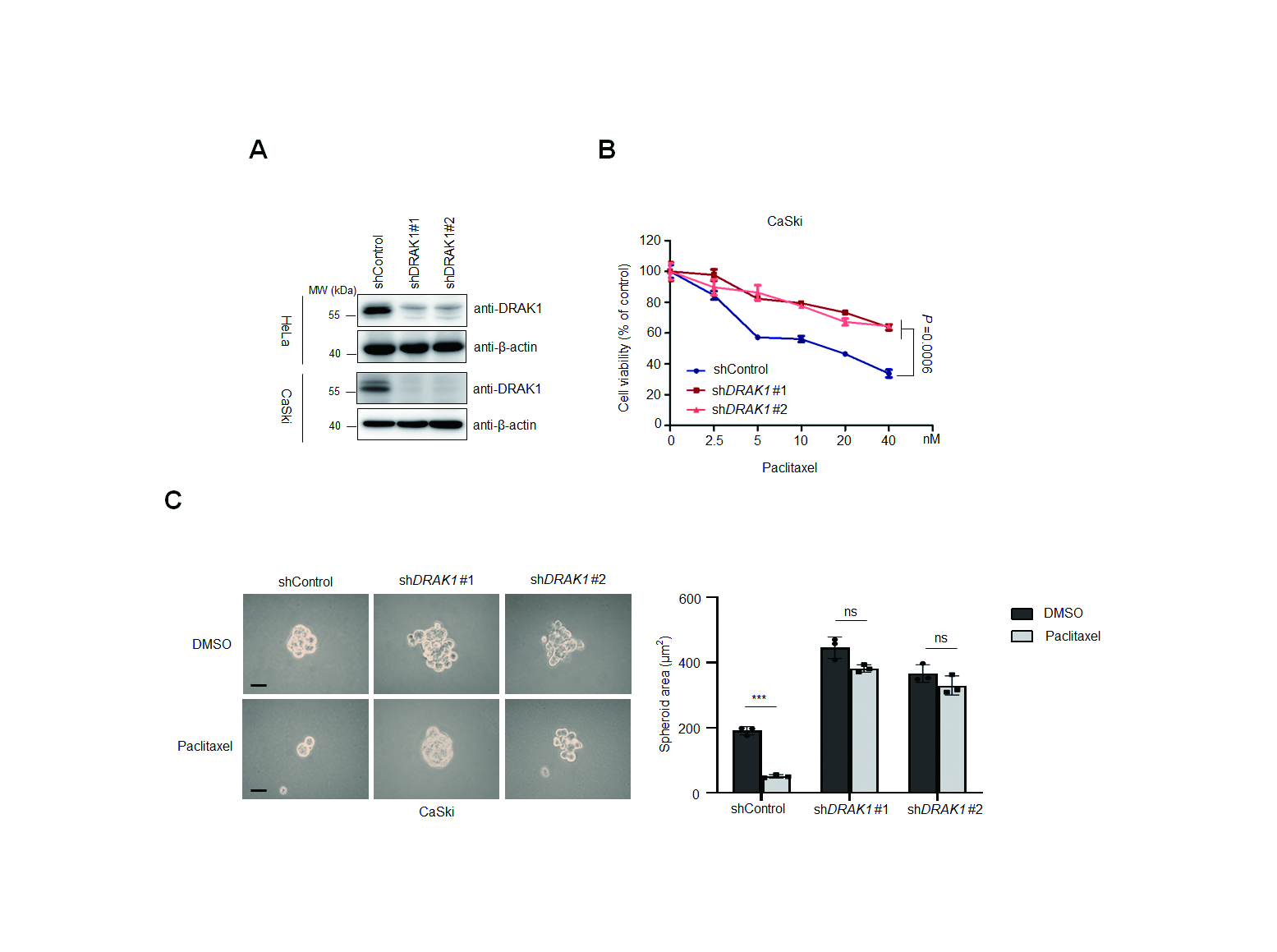

Supplement: Supplementary file 3 — Supplementary Figure S3 [file 41419_2022_4619_MOESM3_ESM.tif]

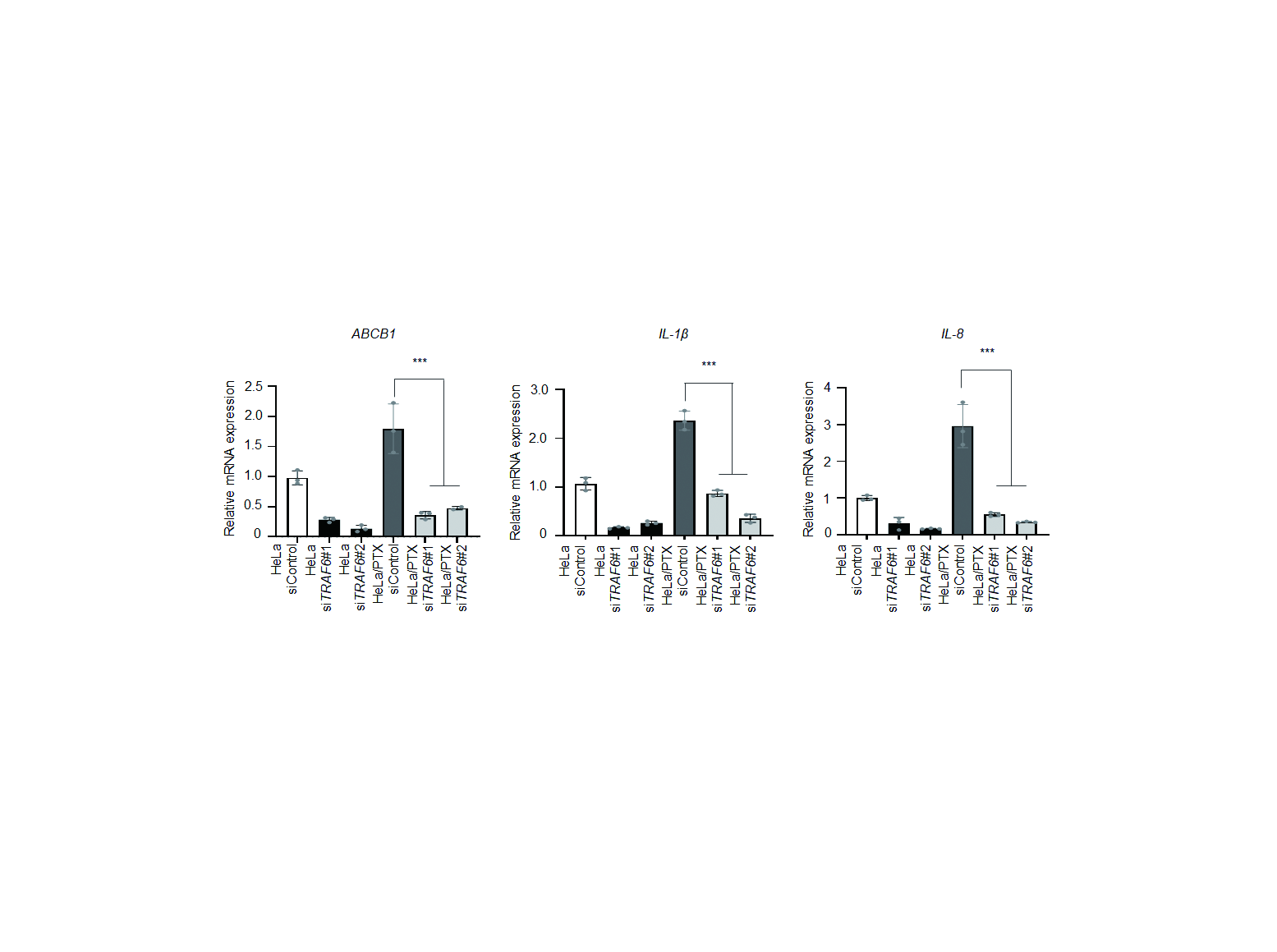

Supplement: Supplementary file 4 — Supplementary Figure S4 [file 41419_2022_4619_MOESM4_ESM.tif]

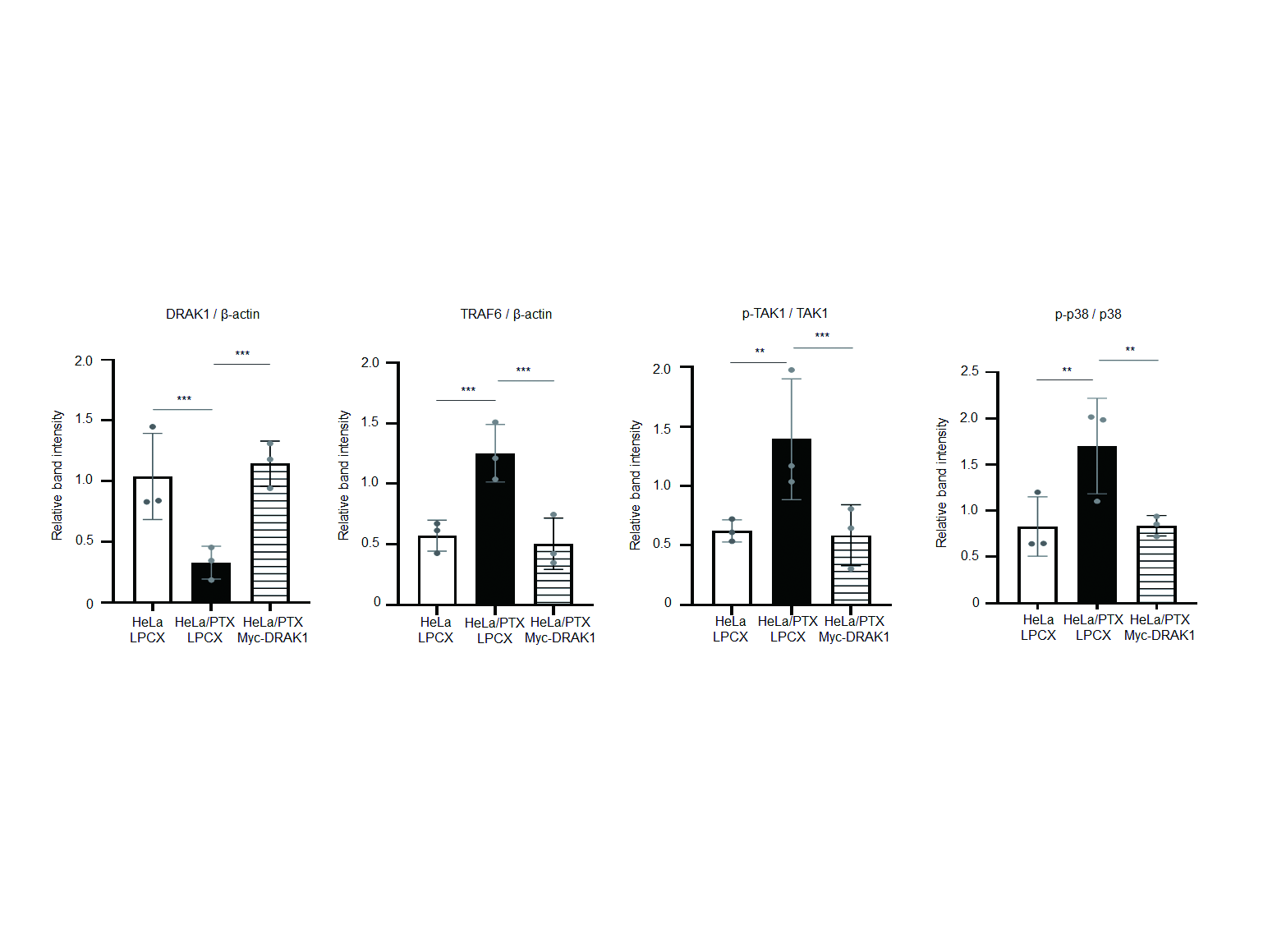

Supplement: Supplementary file 5 — Supplementary Figure S5 [file 41419_2022_4619_MOESM5_ESM.tif]

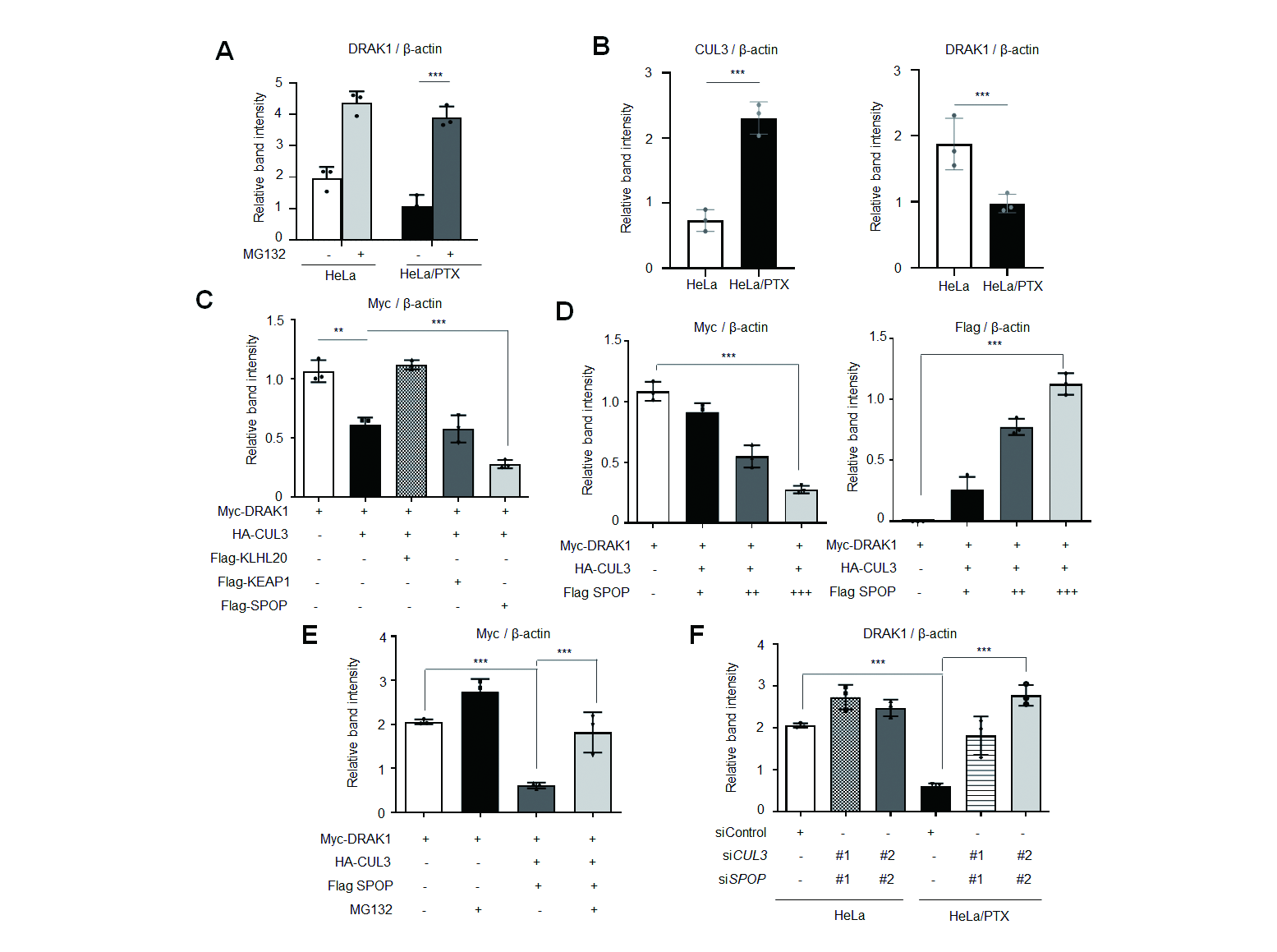

Supplement: Supplementary file 6 — Supplementary Figure S6 [file 41419_2022_4619_MOESM6_ESM.tif]
