# Supplementary material for: Degradation of DRAK1 by CUL3/SPOP E3 Ubiquitin ligase promotes tumor growth of paclitaxel-resistant cervical cancer cells
Source: Cell Death Dis. 2022 Feb 22;13(2):169. doi: 10.1038/s41419-022-04619-w (PMC8863983; doi:10.1038/s41419-022-04619-w)
Supplement: Supplementary file 7 — Supplementary Figure Legends [file 41419_2022_4619_MOESM7_ESM.docx]

Supplementary Information

**Degradation of DRAK1 by CUL3/SPOP E3 Ubiquitin ligase promotes tumor growth of paclitaxel-resistant cervical cancer cells**

Kyoungwha Pang, Jihee Lee, Junil Kim, Jinah Park, Yuna Park, Eunji Hong, Haein An, Akira Ooshima, Kyung-Soon Park, Jae Hyun Cho, [Cheol Lee](https://pubmed.ncbi.nlm.nih.gov/?size=50&term=Lee+C&cauthor_id=32265222) , [Yong Sang Song](https://pubmed.ncbi.nlm.nih.gov/?size=50&term=Song+YS&cauthor_id=32265222) , Kyung-Min Yang, and Seong-Jin Kim

**Supplementary Figure S1** Densitometric quantitation of immunoblot bands for **Fig. 1A**. **P*<0.05, ***P*<0.01, ****P*<0.001 versus control cells. All *P* values were calculated by unpaired two-tailed Student’s *t*-tests. The data represent the mean ± S.D. of three independent experiments.

**Supplementary Figure S2.** NF-κB promoter activity is enhanced in HeLa/PTX cells. Luciferase activity of the NF-κB promoter in parental HeLa and HeLa/PTX cells. *P* values were calculated by unpaired two-tailed Student’s t-tests. Error bars indicate the mean ± S.D. of three independent experiments.

**Supplementary Figure S3.** DRAK1 knockdown increases resistance to paclitaxel in CaSki cells. **A** Immunoblot analysis showing DRAK1 knockdown efficiencies in both HeLa and CaSki cell lines and β-actin was used for internal control. **B** Cell viability assay of *DRAK1*-knockdowned CaSki cells upon paclitaxel treatment ranging from 2.5 to 40nM. Experiments were performed in triplicates. Error bars indicate the mean ± S.D. of three independent experiments. * p<0.05; ** p<0.01; *** p<0.001. **C** Spheroid formation assay of *DRAK1*-knockdowned CaSki cells upon paclitaxel treatment (10nM). Original magnification 100x. Scale bar, 20 μm. Experiments were performed in triplicates. Error bars indicate the mean ± S.D. of three independent experiments. ns = non-significant * p<0.05; ** p<0.01; *** p<0.001.

**Supplementary Figure S4.** NF-κB target genes expression is decreased by the siRNA-induced depletion of *TRAF6* in HeLa and HeLa/PTX cells. qRT-PCR showing the target genes, *ABCB1, IL-1β,* and *IL-8* expression in TRAF6-depleted HeLa and HeLa/PTX cells.

**Supplementary Figure S5.** Densitometric quantitation of immunoblot bands for **Fig. 3A**. **P*<0.05, ***P*<0.01, ****P*<0.001 versus control cells. All *P* values were calculated by unpaired two-tailed Student’s *t*-tests. The data represent the mean ± S.D. of three independent experiments.

**Supplementary Figure S6. A** Densitometric quantitation of immunoblot bands for **Fig. 5A**. **B** Densitometric quantitation of immunoblot bands for **Fig. 5C**. **C** Densitometric quantitation of immunoblot bands for **Fig. 5D**. **D** Densitometric quantitation of immunoblot bands for **Fig. 5E**. **E** Densitometric quantitation of immunoblot bands for **Fig. 5F**. **F** Densitometric quantitation of immunoblot bands for **Fig. 5G**. **P*<0.05, ***P*<0.01, ****P*<0.001. All *P* values were calculated by unpaired two-tailed Student’s *t*-tests. The data represent the mean ± S.D. of three independent experiments.
