# Supplementary material for: Degradation of DRAK1 by CUL3/SPOP E3 Ubiquitin ligase promotes tumor growth of paclitaxel-resistant cervical cancer cells
Source: Cell Death Dis. 2022 Feb 22;13(2):169. doi: 10.1038/s41419-022-04619-w (PMC8863983; doi:10.1038/s41419-022-04619-w)
Supplement: Supplementary file 8 — Supplementary Table 1 [file 41419_2022_4619_MOESM8_ESM.docx]

**Supplementary Table 1. List of primer sequences used in this study**

Primer sequences used to construct plasmids in this study.

| **Construct** | **Direction** | **Sequence (5' - 3')** |
| --- | --- | --- |
| HA-CUL3 | Forward | CCGCTCGAGATGTCGAATCTGAGCAAAGGCACG |
|  | Reverse | CTAGCTAGCTTATGCTACATATGTGTATACTTTG |
| Flag-KLHL20 | Forward | GCCTCGAGATGGAAGGAAAGCCAATGCGCAGGTG |
|  | Reverse | GCGCTAGCTCACCAAATATGGGATTCACAATGTG |
| Flag-KEAP1 | Forward | GCCTCGAGATGCAGCCAGATCCCAGGCCTAGCGG |
|  | Reverse | GCGCTAGCTCAACAGGTACAGTTCTGCTGGTCAA |
| Flag-SPOP | Forward | GCCTCGAGATGTCAAGGGTTCCAAGTCCTCCACC |
|  | Reverse | GCGCTAGCTTAGGATTGCTTCAGGCGTTTGCGT |

The target sequences of siRNAs used in this study

| **Lentiviral shRNA** | **Species** | **Target Sequences** |
| --- | --- | --- |
| sh*DRAK1*#1 | Human | GATTGCTGTACTTGAACTACTC |
| sh*DRAK1* #2 | Human | CAGAGCAGTATTCAAGAGCCTCTC |

| **siRNA** | **Species** | **Target Sequences** |
| --- | --- | --- |
| si*TRAF6 #*1 | Human | CUGUGAAUACUGCAAUACU |
| si*TRAF6* #2 | Human | GUGAGAUUCUUUCUCUGA |
| si*CUL3 #*1 | Human | GUAAACAUGAGCGAUGUAA |
| si*CUL3 #*2 | Human | CAGGUAAUGAUACAGAGAU |
| si*SPOP #*1 | Human | CUCUUGUCCUCCCUGAGUA |
| si*SPOP #*2 | Human | CACAGAUCAAGGUAGUGAA |

Primer sequences for RT-PCR used in this study

| **Construct** | **Species** | **Direction** | **Sequence (5' - 3')** |
| --- | --- | --- | --- |
| *CUL3* | Human | Forward | CACACCAAAGTGCAACATCC |
|  |  | Reverse | AACCCGCTGTGTTGGTTTAC |
| *18S* | Human | Forward | CCCAACTTCTTAGAGGGACA |
|  |  | Reverse | TAGTCAAGTTCGACCGTCTT |

Primer sequences for real-time RT-PCR used in this study

| **Construct** | **Species** | **Direction** | **Sequence (5' - 3')** |
| --- | --- | --- | --- |
| *DRAK1* | Human | Forward | GAAGATCGAGCCACTGCTGA |
|  |  | Reverse | TGACCTTCTTGGAGGGCATTT |
| *TRAF6* | Human | Forward | ACGCCACCTACAAGAGAACA |
|  |  | Reverse | CCAGAGTCGGGTATAACGCT |
| *ABCB1* | Human | Forward | CCTGACTCACCACACCAATG |
|  |  | Reverse | CAGAGGGGATGGTCAGTGTT |
| *IL-1β* | Human | Forward | AATCTGTACCTGTCCTGCGTGTT |
|  |  | Reverse | TGGGTAATTTTTGGGATCTACACTCT |
| *IL-8* | Human | Forward | CTTGGCAGCCTTCCTGATTT |
|  |  | Reverse | TTCTTTAGCACTCCTTGGCAAAA |
| *E4F1* | Human | Forward | AAATCCGCTTCAGTGTGAGC |
|  |  | Reverse | CTATAGGCTCGCCTGTCACC |
| *CCNB2* | Human | Forward | TGCAAAATCGAGGACATTGA |
|  |  | Reverse | TGTGGGTTTATGGACTGCAA |
| *CDK10* | Human | Forward | TTGTCGCACTGAAGAAGGTG |
|  |  | Reverse | CTCCTTCAGCTCCACGATGT |
| *MYC* | Human | Forward | TCAAGAGGCGAACACACAAC |
|  |  | Reverse | GGCCTTTTCATTGTTTTCCA |
| *RBP5* | Human | Forward | TGCAGTTTGATGTGGGAGTG |
|  |  | Reverse | CCTTTCTGCACACACACCAG |
| *BCCIP* | Human | Forward | ATGAGGAGCAGGGAAAACCT |
|  |  | Reverse | TTCAGAGAAACCAGGGCTGT |
| *CCNE2* | Human | Forward | TTGGCTATGCTGGAGGAAGT |
|  |  | Reverse | CCTGGTGGTTTTTCAGTGCT |
| *CINP* | Human | Forward | TTGGATGGGTTGACCAAAAT |
|  |  | Reverse | GAGGGGGTCGTTTACTCTCC |
| *CDC25L* | Human | Forward | TCCTTAAAGGCGGCTACAGA |
|  |  | Reverse | TCGACACCTCAGCAACTCAG |
| *GSTP1* | Human | Forward | AGTGGGTCGTGCCTATTTTG |
|  |  | Reverse | AGCCAAATCAGCTTGAGAGG |
| *18S* | Human | Forward | AATGCTTCTCTGGCACGTCT |
|  |  | Reverse | TCTTCCATCTCACGCATCTG |
