# Supplementary material for: Degradation of DRAK1 by CUL3/SPOP E3 Ubiquitin ligase promotes tumor growth of paclitaxel-resistant cervical cancer cells
Source: Cell Death Dis. 2022 Feb 22;13(2):169. doi: 10.1038/s41419-022-04619-w (PMC8863983; doi:10.1038/s41419-022-04619-w)
Supplement: Supplementary file 9 — Supplementary Table 2 [file 41419_2022_4619_MOESM9_ESM.docx]

**Supplemental Table 2.** List of antibodies used in this study

| Name | Catalog No. | Application | Species | Company |
| --- | --- | --- | --- | --- |
| DRAK1 | NBP1-76896 | Immunoblotting (1:1000) | Rabbit | Novous Biologicals |
| DRAK1 | am20971PU-N | Immunohistochemistry (1:200)  Immunoprecipitation (1:100) | Mouse | OriGene |
| TRAF6 | sc-8409 | Immunohistochemistry (1:100) | Mouse | Santa Cruz |
| TRAF6 | sc-7220 | Immunoblotting (1:1000) | Rabbit | Santa Cruz |
| TAK1 | 5208 | Immunoblotting (1:1000) | Rabbit | Cell Signaling |
| p-TAK1 | 4508 | Immunoblotting (1:1000) | Rabbit | Cell Signaling |
| p38 | 9212 | Immunoblotting (1:1000) | Rabbit | Cell Signaling |
| p-p38 | 9211 | Immunoblotting (1:500) | Rabbit | Cell Signaling |
| CUL3 | sc-166110 | Immunoblotting (1:1000) | Mouse | Santa Cruz |
| SPOP | ab137537 | Immunoblotting (1:1000) | Rabbit | abcam |
| K48-Ub | 33959T | Immunoblotting (1:1000) | Rabbit | Cell Signaling |
| K63-Ub | 33959T | Immunoblotting (1:1000) | Rabbit | Cell Signaling |
| Myc (9E10) | sc-40 | Immunoblotting (1:2000)  Immunoprecipitation (1:100) | Mouse | Santa Cruz |
| HA (F-7) | sc-7392 | Immunoblotting (1:1000) | Mouse | Santa Cruz |
| Flag (M2) | F3165 | Immunoblotting (1:5000) | Mouse | Sigma |
| β-actin (AC-74) | A5316 | Immunoblotting (1:10000) | Mouse | Sigma |
